# PCalign: a method to quantify physicochemical similarity of protein-protein interfaces

**DOI:** 10.1186/s12859-015-0471-x

**Published:** 2015-02-01

**Authors:** Shanshan Cheng, Yang Zhang, Charles L Brooks

**Affiliations:** 1 Department of Computational Medicine and Bioinformatics, Medical School, University of Michigan, Ann Arbor, MI USA; 2 Department of Biological Chemistry, Medical School, University of Michigan, Ann Arbor, MI USA; 3 Department of Chemistry, University of Michigan, Ann Arbor, MI USA; 4 Biophysics Program, University of Michigan, Ann Arbor, MI USA

**Keywords:** Protein-protein interactions, Interface comparison, Structural bioinformatics, Convergent evolution

## Abstract

**Background:**

Structural comparison of protein-protein interfaces provides valuable insights into the functional relationship between proteins, which may not solely arise from shared evolutionary origin. A few methods that exist for such comparative studies have focused on structural models determined at atomic resolution, and may miss out interesting patterns present in large macromolecular complexes that are typically solved by low-resolution techniques.

**Results:**

We developed a coarse-grained method, PCalign, to quantitatively evaluate physicochemical similarities between a given pair of protein-protein interfaces. This method uses an order-independent algorithm, geometric hashing, to superimpose the backbone atoms of a given pair of interfaces, and provides a normalized scoring function, PC-score, to account for the extent of overlap in terms of both geometric and chemical characteristics. We demonstrate that PCalign outperforms existing methods, and additionally facilitates comparative studies across models of different resolutions, which are not accommodated by existing methods. Furthermore, we illustrate potential application of our method to recognize interesting biological relationships masked by apparent lack of structural similarity.

**Conclusions:**

PCalign is a useful method in recognizing shared chemical and spatial patterns among protein-protein interfaces. It outperforms existing methods for high-quality data, and additionally facilitates comparison across structural models with different levels of details with proven robustness against noise.

**Electronic supplementary material:**

The online version of this article (doi:10.1186/s12859-015-0471-x) contains supplementary material, which is available to authorized users.

## Background

Protein-protein interactions play important functional roles in almost all biological activities, including, but not restricted to, signal transduction, gene regulation, catalytic enzymatic activities and structural roles [1]. Characterization and classification of protein-protein interactions would allow us to organize information in protein-protein interaction networks, to make predictions on their function, as well as to facilitate drug design targeted at interfering with those disease-associated protein-protein interactions. Advances in experimental techniques in recent years have led to exponential growth in structural data available for protein complexes [2], and the rise of low-resolution alternative techniques such as cryo-Electron Microscopy (cryo-EM) have made it possible to visualize even large macromolecular complexes that were previously not amenable to crystallization [3].

At the center of protein-protein interactions are the binding surfaces, or interfacial residues which form contacts between binding partners and stabilize protein complexes. Characteristics of residues lining interfaces have been extensively studied, some focusing on their collective statistics such as hydrophobicity, buried surface area, depth index and planarity [4-6], others focusing on hot spot residues which contribute significantly to the free energy of binding [7,8]. While these approaches provide insights into the mechanism of protein-protein recognition, they are not suitable for measuring similarities between a given pair of protein-protein interfaces. The latter is useful for revealing potential biological relationships between different complexes, and a suitable method to directly compare protein-protein interfaces across randomly selected protein complexes and to quantitatively assess their pairwise similarities is highly desirable.

Depending on the specific biological question being asked, methods for protein-protein interface comparison with different focuses have been developed. Galinter [9], for one, finds alignment for van der Waals and hydrogen bonding interactions at an interface, but does not provide a quantitative measure of interface similarity. Gao and Skolnick developed the dynamic programming-based algorithm Ialign [10] to detect protein-protein interfaces with shared geometric patterns. The currently available implementation for this method initially defines interfacial residues using atomic details, and then uses positions of the Cα atoms for structural alignment and scoring, and includes a sequence-order dependent version [10] and a sequence-order independent version [11]. Shulman-Peleg *et al.* developed I2I-SiteEngine [12] to compare the physicochemical properties of the functional groups forming protein-protein interfaces, which uses an algorithm similar to pharmacophore mapping. An extension of this same algorithm, MAPPIS [13], has also been developed for aligning multiple protein-protein interfaces simultaneously. Although both Ialign and I2I-SiteEngine work well for accurately predicting functional relationships between protein complexes determined at atomic resolution, they require high resolution of the structural models in order to determine what constitutes an interface. As a result, they are not applicable in cases where the data quality of structural models is relatively poor and only backbone atoms are traceable, which frequently correspond to large macromolecular complexes such as viral capsid shells.

Given that there may be protein-protein interaction patterns that occur exclusively in large oligomeric complexes, there is significant added value in exploring these coarse-grained structural models when studying protein-protein interfaces, calling for an interface comparison method that can traverse through different resolutions of structural models.

In this work, we develop a method that combines the advantages of existing methods to quantify the similarity of any given pair of protein-protein interfaces in terms of their physicochemical properties. This method not only disregards the sequence-order of interface fragments in performing the structural alignment, but also takes into account the mapping of different chemical types of amino acid residues. More importantly, our method facilitates comparison of structural models determined at different resolutions, greatly expanding the structural space of protein-protein interactions that can be studied systematically.

## Methods

### Extract interfacial residues

While many structural models for protein monomers and homodimers can be determined at atomic resolution by X-ray crystallography or NMR techniques, the structures of large macromolecular complexes are typically solved by cryo-EM and are hence low in resolution. In spite of their lower resolution, these coarser structural models are nonetheless informative and can aid our understanding of protein-protein interactions. Statistics from PDB show over 600 structural models only contain information on the Cα atoms, with many of them populating the lower end of the resolution spectrum (Figure S1 in Additional file 1). To facilitate comparison of interfaces across models with different levels of details, we apply a hierarchical approach in defining interfacial residues with a distance criterion, as described below.

Given the structural model of a protein dimer determined at atomic resolution, we define two residues to be in contact if at least two heavy atoms, one from each residue, are within 4.5 Å. The collection of all residues that are in contact with at least one residue in the binding partner is considered the set of interfacial residues. When side chain information is not available, we use a Cα-Cα distance cutoff criterion to determine if two residues are in contact. Traditionally, a common distance cutoff is used for all types of amino acids. Considering the fact that different amino acids have side chains that vary in size, the Cα-Cα distance between different types of pairs of amino acids that make a contact via their side chains may differ by a non-negligible amount. To account for the side chain size factor, we examined the statistics of Cα-Cα distances for different pairs of amino acids (e.g., a Ser-Lys pair) that form intermolecular contacts from PDB. Similar to what Kolinski and Skolnick did in parameterizing pairwise interactions between side chain groups of different amino acids [14], our distance cutoff for Cα-Cα distances is determined by the following,

1 $$ {\mathrm{cutoff}}_{i,j}=\kern0.5em {\mathrm{mean}}_{i,j}+\upxi \kern0.5em \times \kern0.5em {\mathrm{sd}}_{i,j} $$

where *i*,*j* represent a given pair of residues of amino acid type *i* and of amino acid type *j* respectively (*i*, *j* can be the same type). Mean_*i*,*j*_ and sd_*i*,*j*_ represent the average values of the Cα-Cα distances in the set of high-resolution (37474) structures in the PDB and their standard deviation respectively. The statistics are listed in Additional file 1: Table S1a, S1b. ξ represents a multiplication factor that is of a fixed value across different residue types, and its optimal value was determined to be 0.5 based on our correlation study (see Additional file 1: Figure S2).

After extraction of the interfacial residues, only the coordinates of the Cα atoms are retained for use in computing the pairwise interface similarity score, so as to allow comparison of interfaces with different levels of structural details. This bare bones criterion sufficiently captures the skeleton architecture that hosts amino acid residues of various chemical types at an interface, without adding noise to the data representation that arises from fluctuations of the side chain orientations.

### Identify initial alignments

In order to quantitatively assess the degree to which two sets of interfacial residues resemble each other spatially and chemically, we first superimpose the two sets of interfacial residues. As interfacial residues are fragments that are clustered at the binding site without these fragments necessarily following the peptide sequence order, we chose the sequence order-independent comparison technique, geometric hashing [15-18], to find the transformation needed to superimpose one interface onto the other. This algorithm treats each interface as a set of color-labeled points (of the Cα atoms) scattered in the three-dimensional space, where the color corresponds to the chemical type of each residue. The goal is to find a transformation (i.e. translation and rotation) applied to one point cloud to be overlaid with the other so as to maximize the number of points that match spatially and chemically. Details of the algorithm dealing with simply the geometric properties of the interfacial residues are explained in the Additional file 1. In short, the algorithm uses a voting procedure to count the number of points that can be matched between two sets of points for a given superposition, where a point *i* is described by a feature defined by the Cartesian coordinates of the point, (*x*_*i*_,*y*_*i*_,*z*_*i*_). The superposition that receives a high vote corresponds to one having many points that can be matched between the two sets of interfacial residues.

There are two additional factors to be taken into account in this problem; first is that an interface is not a single entity (a set of points) but rather consists of two binding fragments, A and B. Thus in aligning one interface to another, one needs to simultaneously align fragment A in interface 1 to fragment A’ in interface2, and B to B’, and not allow crossing over. To achieve this, we add to the feature (xyz-coordinates originally) an additional attribute of fragment label (either binding site 1 or binding site 2), and count the votes only when both the coordinates and the fragment labels match. Without knowing the correspondence of binding fragments prior to alignment, we attempt both ways by swapping the fragment labels.

In addition to the fragment label, another factor to be considered is the chemical label that is associated with each residue. Given the coarse-grained nature of our method (using only Cα atoms), we applied a reductive method to classify the 20 amino acids based on the prominent functional group in each side chain. The assignment of the individual functional groups in the side chain is based on the definition in earlier work by Schmitt *et al.* [19]. Depending on what functional groups are present in a specific amino acid, we classified them to one of the following six categories: donor (K, R), acceptor (E, D), mixed donor/acceptor (N, Q, S, T), aromatic (F, W), Aliphatic (C, A, I, L, M, P, V, G) and mixed donor/acceptor or aromatic (H, Y). This classification scheme largely agrees with previous assignment of the residue type [20]. Since the six classes are not mutually exclusive, we allow matches across two different classes as long as they share at least one common functional group. For instance, Asparagine has both a donor group, ND2-HD21, and an acceptor group, OD1, and thus classified as “mixed donor/acceptor”. Therefore Asparagine can be matched with either Arginine due to the shared functional group of a donor, or Aspartate due to the shared functional group of an acceptor. In summary, we consider features representing residues as equal if their binned xyz-coordinates and fragment labels are the same and their chemical labels match (not necessarily identical).

Lastly, different pairs of orthogonal bases typically yield degenerate transformation matrices, and thus those receiving sufficiently high votes are further clustered to retain representative transformations as a last step, with the 100 top-ranking transformations processed for further refinement.

### Iterative refinement

The previous step proposed candidate transformations to superimpose the two interfaces initially, and in the iterative refinement step we aim to further improve the structural alignment in order to maximize the final similarity score. Based on each proposed initial alignment, a list of structurally equivalent pairs of residues between the two interfaces can be identified. Here we used maximum weight matching in bipartite graphs [21] to identify structural equivalence, which is the problem of optimizing one-to-one mapping between two sets of nodes based on the weight of the edge that connects two nodes (one from each set). We implemented the Hungarian algorithm [22], which is explained in detail in the Additional file 1.

In our particular problem, we’d like the weight to reflect spatially how close two residues are and also how well their chemical types match, and hence we’ve chosen the following scheme to quantify equivalence between *i* ^th^ residue in interface 1 and *j* ^th^ residue in interface 2,

2 $$ \mathrm{equivalence}\hbox{-} {\mathrm{score}}_{ij}=\frac{1}{1+0.25\times \left(1\hbox{-} {I}_{ij}\left(\mathrm{same}\kern0.5em \mathrm{chem}\kern0.5em \mathrm{type}\right)\right)+{\scriptscriptstyle \frac{{d_{ij}}^2}{16}}} $$

where *I*_*ij*_(same chem type) is the indicator function that takes the value of 1 when the pair of residues (*i*, *j*) share the same chemical type and 0 when they don’t. *d*_*ij*_ is the Euclidean distance between the Cα atoms of the two residues in Å after structural superposition. After obtaining the list of equivalent residues, we then apply the Kabsch algorithm [23] to translate and rotate the second interface so as to minimize the sum of squared errors between all the equivalent pairs of residues. Based on this new structural superposition, we obtain a new updated list of structurally equivalent residues using maximum weight matching in bipartite graphs, which will be submitted to the same procedure for refinement. This process is iterated until no further improvement is possible.

The overall scoring function, PC-score_raw_, based on the converged alignment is given by

3 $$ \mathrm{P}\mathrm{C}\hbox{-} {\mathrm{score}}_{\mathrm{raw}}=\frac{f_c}{L_{ave}}{\displaystyle {\sum}_{i=1}^{L_{ali}}\frac{1}{1+0.25\times \left(1-{I}_{ii}\left( same\kern0.5em  chem\kern0.5em  type\right)\right)+\frac{{d_{ii}}^2}{16}}} $$

where *L*_ave_ is the average number of interfacial residues for the pair of interfaces compared, and *L*_*ali*_ is the number of all aligned residues identified by the aforementioned algorithm that have an equivalence-score of 0.20 or higher. *f*_c_ is the ratio of common contacts between the two sets of aligned interfacial residues, and is calculated as

4 $$ {f}_c=\frac{{\overleftrightarrow{N}}^1\cdot {\overleftrightarrow{N}}^2}{0.5\times \left({\overleftrightarrow{N}}^1\cdot {\overleftrightarrow{N}}^1+{\overleftrightarrow{N}}^2\cdot {\overleftrightarrow{N}}^2\right)} $$

where ${\overleftrightarrow{N}}^1$ and ${\overleftrightarrow{N}}^2$ are *L*_*ali* ×_ *L*_*ali*_ matrices representing the contact maps of the aligned interfacial residues in interface 1 and those in interface 2 respectively. The dot operation represents inner product. This scoring function is largely adapted from the scoring function of IS-score for the program Ialign [10], given its demonstrated excellent performance in the original study, with the modifications here to specifically address our question of interest.

Finally, the raw PC-score is further scaled by the following equation to remove the dependency of the score on the interface size to derive our final scoring function:

5 $$ {{\mathrm{PC}\hbox{-} \mathrm{score} = \mathrm{P}\mathrm{C}\hbox{-} \mathrm{score}}_{\mathrm{raw}}}^{ln0.3/ In\left(0.14\kern0.5em  + 0.29 \times 0{.97}^{L_{ave}}\right)} $$

This scaling function was derived by fitting the curve of the raw PC-score as a function of the size of randomly selected pairs of interfaces taken from a non-redundant representative data set derived from PDB (see Additional file 1: Figure S3).

The candidate alignment that receives the highest PC-score represents the optimal alignment solution, and its associated PC-score gives the measurement of physicochemical similarity between the two interfaces being compared. This scoring function is normalized between 0 and 1, and takes the value of 1 when comparing two identical interfaces. The associated statistical significance with a PC-score is derived empirically from the distribution of PC-scores for random interface alignments (Additional file 1: Figure S4).

## Results

### Validation of the scoring function

Although the quantification of protein-protein interface similarity has no corresponding experimental observables to benchmark against, we can nonetheless evaluate whether the scoring function is reasonable by comparing it with other physically sound metrics. This is only a proof of concept, but still provides useful information in terms of judging the performance of the method. Specifically, we tested our scoring function against the Q-score in quasi-equivalent viral capsid protein-protein interfaces.

The Q-score is a normalized score based on equivalent residue contacts in interfaces formed by viral capsid proteins [24]. Viral capsid proteins are special structural proteins; repeating units of the same capsid protein assemble into large, symmetric shells that embed the viral genetic materials inside. An inter-subunit interface within a capsid is thus formed by two monomers with the same peptide sequence. In the smallest icosahedral viruses, 60 copies of the same protein tile the icosahedral shell, where each protein is placed in the same environment. Correspondingly, all the interfaces with the same dimerization states are chemically identical. For larger viruses, however, multiples (with the multiplicity denoted by the Triangulation number or T-number) of 60 copies of the same protein assemble into macromolecular complexes that also obey icosahedral symmetry. Based on the theory proposed by Caspar and Klug [25], this can be achieved by allowing slightly varied modes of interaction in the proteins such that those protein-protein interfaces following strict 2-fold, 3-fold or 5-fold symmetry (as in an icosahedron) and those which do not are quasi-equivalent to each other, but not identical.

The Q-score was developed to specifically quantify the level to which two quasi-equivalent interfaces resemble each other. First the contact map represented by an N × N matrix of 1’s and 0’s between the two binding partners of each interface is calculated, where N is the number of amino acids in the capsid protein. The Q-score is computed by taking the normalized inner product of the two contact maps of the interfaces:

6 $$ \mathrm{Q}\hbox{-} \mathrm{score}=\frac{2\times {\overleftrightarrow{N}}^{\mathrm{a}}\cdot {\overleftrightarrow{N}}^{\mathrm{b}}}{\left({\overleftrightarrow{N}}^{\mathrm{a}}\cdot {\overleftrightarrow{N}}^{\mathrm{a}}+{\overleftrightarrow{N}}^{\mathrm{b}}\cdot {\overleftrightarrow{N}}^{\mathrm{b}}\right)} $$

7 $$ =\frac{2\times {\displaystyle {\sum}_i{\displaystyle {\sum}_j{N}_{i,j}^a{N}_{i,j}^b}}}{{\displaystyle {\sum}_i{\displaystyle {\sum}_j\left[{\left({N}_{i,j}^a\right)}^2+{\left({N}_{i,j}^b\right)}^2\right]}}} $$

Where ${\overleftrightarrow{N}}^{\mathrm{a}}$ and ${\overleftrightarrow{N}}^{\mathrm{b}}$ are matrices representing the contact maps of interface a and interface b respectively.

Hence the Q-score reflects the ratio of common contacts between two interfaces, and is equal to 1 for identical interfaces and 0 for two interfaces with no common contacts. This quantification metric is thus a reasonable one with straight forward physical interpretation in the case of capsid protein-protein interactions. We therefore attempted to compare our interface similarity score with the published Q-scores for 18 T = 3 viruses in [24].

Our results show that our interface similarity score largely agrees with the Q-score in viral capsid protein-protein interfaces, with a high overall correlation coefficient of 0.93 (Additional file 1: Table S4). Although the Q-score only measures geometric properties, we showed that with the chemical type taken into consideration in our method, the two agree well, suggesting that our scoring metric is reasonably accurate in capturing the physical properties of interfaces.

### Comparison of performance with existing methods

The performance of PCalign is compared with two existing methods, Ialign with its sequence-order independent version and I2I-SiteEngine [12], by testing how well these methods can distinguish highly similar interfaces from less similar ones. We manually collected from the database, Structural Classification Of Protein-Protein Interfaces (SCOPPI) [26], a set of interfaces which are grouped into several different families based on their evolutionary relationship, and evaluate if our scoring function, as well as the two existing methods, can provide a reasonable cutoff value to separate interfaces that are highly related from those that are not, where the “relatedness” label is given by pairs of interfaces annotated to be in the same group. We should highlight here that the goal of our method is to quantify the physical and chemical properties of protein-protein interfaces, regardless of the familial relationship between the monomers forming the interfaces. Thus benchmarking similarity of interfaces against evolutionary relatedness of the monomers forming the interfaces does not accurately reflect the performance of the three methods. Nonetheless, it gives us a crude measurement of how confident we can be in applying our method to study interfaces in general.

Together we collected 609 dimers from 124 pairs of protein families in SCOPPI, where dimers within the same group are obtained after applying a 50% sequence redundancy filter on the interacting proteins in the database, and are also selected to have similar interaction modes (i.e., they present the same “faces” at the binding sites, based on the database classification) [26]. The 124 groups of pairwise interacting proteins were chosen such that no protein chains across different groups share the same structural fold, where fold is annotated by the first letter and the succeeding number in their SCOP ID (Additional file 1: Table S5). This ensures that the constituent protein monomers across different groups are structurally dissimilar. Together there are 480 homodimers covering 97 groups and 129 heterodimers covering 27 groups in our data set. We then performed an all-against-all comparison for these 609 interfaces using the three methods, and obtained their respective scores for each pair compared. For I2I-SiteEngine, we included all three scores reported by the program, including the match score, the total score, and the t-score [12]. In addition to applying our method to the original data, in order to demonstrate the robustness of our method in application to noisy low-resolution models, we applied the same analysis described above to the “backbone” set, which is the same data set reported here except that all structural models were first “corrupted” to retain their Cα atoms only, and then had the positions of the Cα atom perturbed in a random direction by a magnitude that follows a Gaussian distribution centered at 0 with a standard deviation of 1 Å. This creates an artificial low-resolution data set for testing the robustness of our method. In the Additional file 1, we describe another way of generating a low-resolution data set, which gave similar results to the low-resolution data set in the main text. The two existing methods do not deal with low-resolution data and are thus not applied to this backbone dataset. For any given cutoff value of each score, we tabulated the counts of true positives (TP), false negatives (FN), false positives (FP) and true negatives (TN) for all pairs of interfaces, where real positives correspond to pairs of interfaces belonging to the same pair of protein families, and predicted positives correspond to pairs of interfaces that have a similarity score higher than the given cutoff value. For instance, TP refers to a pair of protein-protein interfaces (each from the 609 dimers) that are evolutionarily related, as annotated to be in the same group by SCOPPI (Additional file 1: Table S5), and at the same time predicted to be similar by a structural comparison method based on a given similarity score by the same method, and FN refers to a homologous pair that falls below a similarity score by a structural comparison method, which regards the pair as dissimilar. We thus derived the receiver operating characteristic (ROC) curve of the three methods so as to evaluate if the method serves as a good classifier of related/unrelated interfaces.

**Figure 1:**
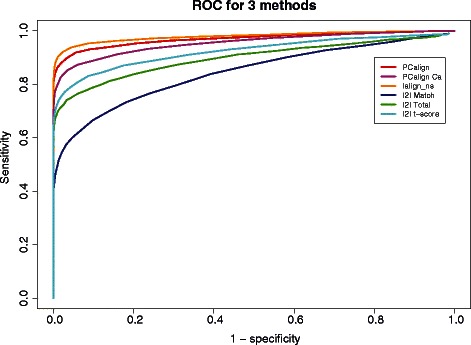
**The ROC curves for predicting highly related interfaces using three methods, PCalign, Ialign, and I2I-SiteEngine.** As shown by the red and magenta curves, our method PCalign gives an AUC value of 0.970, and for the backbone set 0.955. In comparison, Ialign gives an AUC of 0.980. I2I-SiteEngine performs slightly worse, with those predicted by match score, total score and t-score having AUC values of 0.831, 0.884 and 0.909 respectively.

Our results show that our method performs comparably to the two existing methods in capturing most of the interface similarity reflected in the evolutionary relationship. As shown in Figure 1, the area under the curve (AUC) value of predictions derived from IS-score (in orange), which corresponds to the alignment program Ialign, ranks the highest at 0.980. Following Ialign, our PCalign applied to the original data (in red) and to the backbone data (in magenta) resulted in AUC values of 0.970 and 0.955 respectively, which shows that limitation in data quality does not affect the predictive power of our method. The three scores reported by I2I-SiteEngine, the match score (in blue), the total score (in green), and the t-score (in cyan), gave an AUC value of 0.831, 0.884 and 0.909 respectively. Aside from the total benchmark data set, the same analysis applied to the data set partitioned by homodimers and heterodimers shows that all three methods perform better on the heterodimer set than on the homodimer set (see Additional file 1), probably due to the lower likelihood of two different binding fragments in the case of heterodimers to be structurally similar simultaneously for non-homologous pairs, and consequently higher power by the three methods to discriminate against them. Overall, we see that Ialign is a very useful tool in predicting highly related interfaces. Although our method may appear inferior when applied to this data set, it should be noted that our method is developed to detect similar interfaces, which may result not only from evolutionary relatedness but also arise from nature’s recycling her limited choices of interface design. What were reported by PC-score to be FPs could well be putative positives based on interface and not monomer structural similarity, which can be captured by our program and may be dismissed by Ialign. To verify if indeed this is the case, we carried out further analysis as follows.

**Figure 2:**
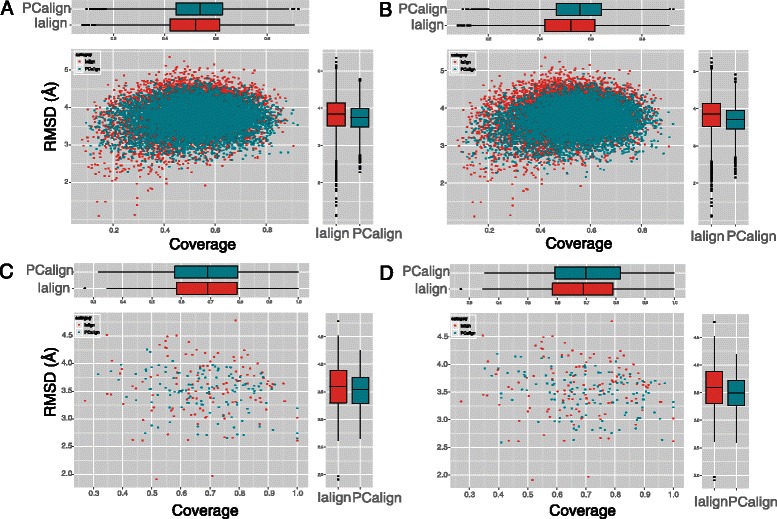
**Recognition of interface similarity across unrelated interfaces by PCalign and Ialign.** The comparison is based on two geometric criteria; fraction of aligned residues (coverage) and RMSD of aligned residues. **(A)** All-against-all pairwise comparison, with PCalign (Ialign) aligning on average 53.1 ± 13.3% (51.4 ± 13.8%) of residues with RMSD of 3.725 ± 0.371 Å (3.810 ± 0.473 Å). **(B)** All-against-all pairwise comparison, with PCalign (Ialign) aligning on average 54.8 ± 13.2% (51.4 ± 13.8%) of residues with RMSD of 3.686 ± 0.378 Å (3.810 ± 0.473 Å), where the chemical term in PCalign is turned off to capture geometric similarity only. **(C)** Closest unrelated interface in the set of 124 dimers, with PCalign (Ialign) aligning on average 68.4 ± 14.5% (68.3 ± 15.6%) of residues with RMSD of 3.483 ± 0.366 Å (3.563 ± 0.502 Å). **(D)** Closest unrelated interface in the set of 124 dimers, with PCalign (Ialign) aligning on average 70.1 ± 15.4% (68.3 ± 15.6%) of residues with RMSD of 3.466 ± 0.371 Å (3.563 ± 0.502 Å), considering the geometric part of the scoring function in PCalign only. In all scenarios, PCalign does slightly better than Ialign in recognizing geometric similarities across unrelated interfaces, and using a scoring function that considers both chemical and geometric properties in PC-score performs less well compared to using one that considers purely geometric properties in PC-score, due to the fact that this analysis uses purely geometric criteria.

Because we are interested in knowing if PCalign does better than Ialign in recognizing interface similarity across unrelated protein dimers, we select one representative structure from each of the 124 clusters of protein dimers, and compare all-against-all. For each pair compared, we tabulate the fraction of aligned interfacial residues (i.e. coverage) as well as the RMSD between the aligned interfacial residues. Ideally, a good structural alignment program should find high coverage and low RMSD values. Note that this criterion is purely geometric. As shown in Figure 2A, PCalign aligns slightly more interfacial residues on average compared to Ialign, with lower RMSD as well, even though PCalign considers both geometric and chemical aspects of protein-protein interfaces. When the chemical term, 0.25 × (1–*I*_*ii*_ (same chem type)), in our scoring function (Equations 2 and 3) is turned off, the advantage of PCalign over Ialign becomes more pronounced (Figure 2B). In addition, we find for each of the 124 interfaces its nearest neighbor in the non-redundant set based on PC-score and IS-score respectively, and perform the same analysis. We again observe the same trend for the closest, unrelated match identified by the two methods, with PCalign outperforming Ialign with and without the chemical term considered (Figure 2C,D). While apparently the improvement in finding a better structural alignment across unrelated interfaces by PCalign compared to Ialign seems marginal on average, our paired Wilcoxon test performed on the data set in Figure 2A shows otherwise, with PCalign having statistically significant higher coverage (p = 2.552E-15) and lower RMSD (p < 2.2E-16) compared to Ialign.

**Figure 3:**
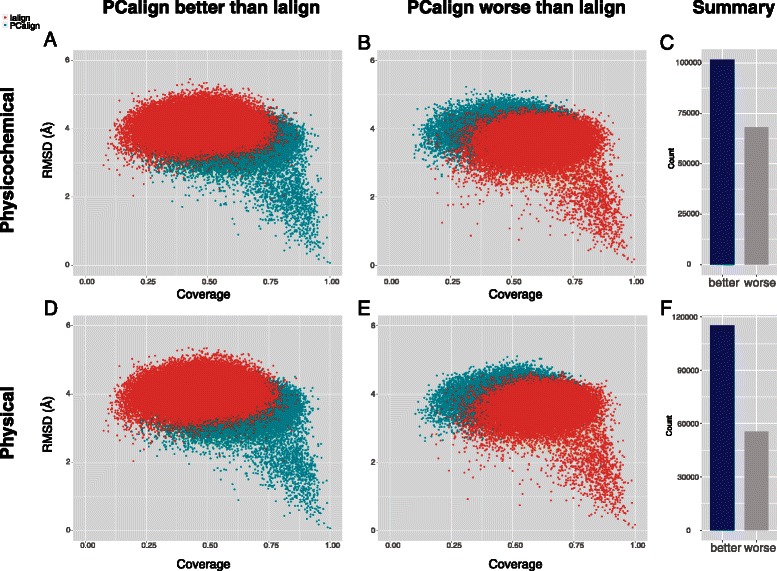
**Further performance comparison between PCalign and Ialign.** When a method finds higher coverage with lower RMSD for a particular pair of interfaces compared, it is considered better for that case; with lower coverage and higher RMSD, it is considered worse. All cases of PCalign (plotted in green) outperforming Ialign (plotted in orange) and Ialign outperforming PCalign in the 185136 pairs compared are shown in the scatter plots and summarized in the bar plots. **(A)** In 50838 cases, PCalign is better than Ialign, when the chemical term in PC-score is turned on. **(B)** Ialign outperforms PCalign in 34087 cases in comparison. **(C)** PCalign has an odds ratio of 1.5 in finding a better structural alignment than Ialign. **(D)** When only geometrical property is considered in the scoring function, PCalign outperforms Ialign in 57639 cases. **(E)** Ialign is better than PCalign in 27790 cases. **(F)** PCalign outperforms Ialign with an odds ratio of 2.1 if only the physical environment of protein-protein interfaces is considered.

As such, our method may detect more often than other methods highly similar but non-related interfaces that are counted as FPs in this benchmark test, which is the question of interest that our method aims to address and which cannot be accurately assessed by the classification analysis in Figure 1. To further check the odds of our method outperforming Ialign versus the other way round in terms of getting a better structural alignment, we tabulate the statistics of each scenario among all 185136 pairs compared, again using the same geometric criterion as in Figure 2. With the chemical term switched on, PCalign outperforms Ialign in 50838 pairs, with an average coverage of 0.623 and an average RMSD of 3.64 Å, as compared to 0.483 and 4.10 Å for Ialign respectively (Figure 3A). Ialign is found to outperform PCalign in 34087 cases, with an average coverage of 0.619 and an average RMSD of 3.60 Å, as compared to 0.486 and 4.04 Å for PCalign (Figure 3B). If we remove the compounding factor of chemical types for fairer comparison, PCalign outperforms Ialign in 57639 cases, with an average coverage of 0.629 and an average RMSD of 3.63 Å, as compared to 0.486 and 4.09 Å for Ialign respectively (Figure 3D). In contrast, Ialign does better than PCalign in only 27790 cases, with an average coverage of 0.630 and an average RMSD of 3.56 Å, as compared to 0.504 and 3.99 Å for PCalign (Figure 3E). In summary, we have an odds ratio of 1.5 for PCalign doing better than Ialign with the original scoring function of PCalign (Figure 3C), and an odds ratio of 2.1 for PCalign performing better when we only consider the physical environment of protein-protein interfaces (Figure 3F). Figure S5 in the Additional file 1 gives an anecdotal illustration of a scenario where PCalign recognizes significant structural similarity between two unrelated interfaces that is missed by Ialign.

In terms of computational time, our method is slightly slower by less than an order of magnitude compared with Ialign and faster than I2I-SiteEngine (Additional file 1: Figure S6). This higher computational cost arises from the algorithm complexity, which samples a larger initial alignment space by disregarding the peptide topology. Nonetheless, such cost is sufficiently low for our method to be applied to large-scale comparison studies. We would therefore argue that the performance of our method parallels those of existing methods. Additionally, our method tackles structural models spanning the resolution spectra, which existing methods fail to do, and is able to detect spatial and chemical patterns shared by interfaces regardless of their sequence similarity in the constituent monomers. We thus expect our method to be a handy tool in exploring the repertoire of protein-protein interfaces and understanding their structural relationships.

### Application of the method in detecting convergently evolved similar interfaces

Our method quantifies interface similarity based on the spatial and chemical organization of discontinuous interface fragments. This method therefore accounts for sequence order-independent patterns shared between protein-protein interfaces that arise not necessarily from divergent evolution, but potentially from convergent evolution, which leads to identification of functional relationships masked by apparent lack of structural resemblance.

One such interesting example is viral mimicry. Over the long-standing history of pathogen-host interaction, viruses have evolved various strategies to evade detection by the host immune system [27], to manipulate the cellular signaling network to their advantage [28], and to hijack the cellular transcription and translation machinery for self-replication [29]. Among these strategies is molecular mimicry, which can arise sometimes from viruses capturing host genes followed by deriving their homologues via divergent evolution, and more frequently from viruses independently evolving similar binding sites without any sequence or structural similarity to the endogenous protein they compete with [30]. The latter is especially of interest, as being able to identify which endogenous proteins are displaced by these viral proteins when such mimicry is masked by the lack of sequence and structural similarity can significantly enhance our understanding of how viruses interfere with the cellular pathways for their purposes.

**Figure 4:**
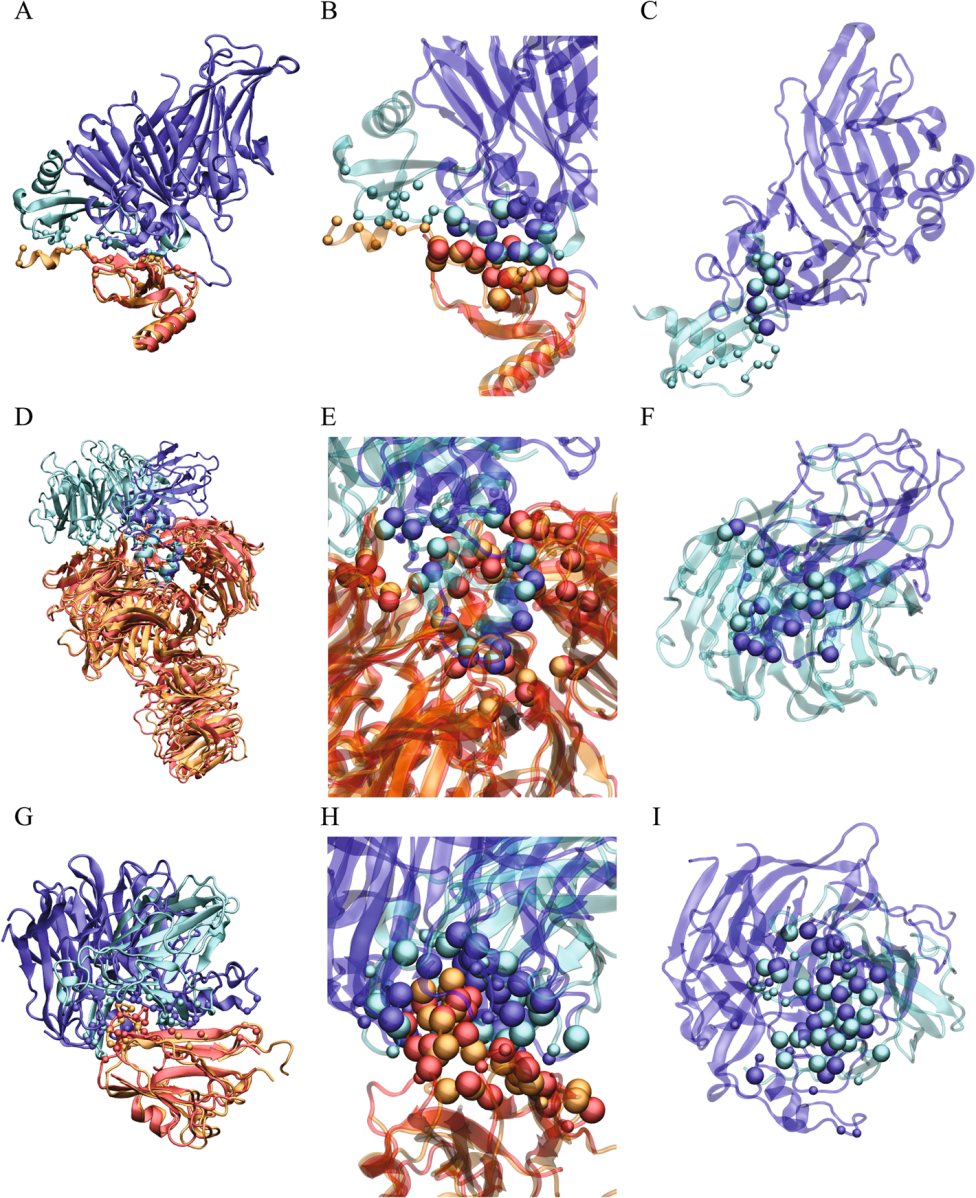
**Three examples of viral mimicry resulting from convergent evolution.** The first example is that of the M3 protein mimicking CCL2 in complexing with another CCL2 monomer **(ABC)**, the second being the V protein competing with DDB2 in binding with DDB1 **(DEF)**, and the third case being the G protein targeting the ephrin B2 ligand in similar ways with its native ephrin type-B receptor 4 **(GHI)**. They are shown with the two complexes superimposed **(ADG)**, with a focused view of the matched interfacial residues **(BEH)**, and with just one binding site on the viral protein and that on the host protein it mimics **(CFI)**. In all illustrations the viral protein is colored in blue, and the host protein it displaces is colored in cyan. The human target protein is colored red when bound with the viral protein, and orange when complexed with its cognate binding partner. The small spheres represent the Cα positions of all the interfacial residues present in the original complex, while the large spheres represent those which are structurally equivalent in the virus-host protein complex and in the endogenous complex. Figures are generated by the VMD software [35].

To illustrate the usefulness of our tool in detecting interface mimicry in virus-host interaction, we show here three examples of viral mimicry that are well understood (Additional file 1: Table S6). The first example concerning immune evasion is that of the Murid herpesvirus 4 M3 protein, which binds strongly to the CC chemokine ligand 2/monocyte chemoattractant protein 1 (CCL2/MCP-1) [31]. It is known that chemokines play a crucial role in inducing directed chemotaxis for trafficking of nearby leukocytes [32], which is part of the host immune response. Studies have shown that oligomerization of the CC chemokines, among other types of chemokines, is critical for recruiting cells *in vivo* [33]. Herpesvirus thus evolved the M3 protein as a decoy receptor for CCL2, which binds strongly to the chemokine at the same site where it forms a homodimer with another chemokine [34], therefore inhibiting oligomerization of chemokines that is necessary for its recruitment of leukocytes. Using our method PCalign, we found that despite the complete lack of sequence and structural similarity between the viral protein and the one it displaces, the M3-CCL2 interface indeed overlaps extensively with that of the CCL2 homodimer interface, with a high PC-score of 0.445 (Figure 4A, 4B, 4C and the corresponding VMD files are provided in Additional file 2 of supplementary material).

In a second case pertaining to viral pathogenesis, the Simian virus 5 V proteins target the DNA damage-binding protein 1 (DDB1), a protein involved in the ubiquitin-proteasome pathway, leading to degradation of the STAT1 protein [36]. The latter results in the type I interferon signaling pathway being blocked, effectively preventing the establishment of a cellular antiviral environment [37]. In achieving this function, the viral protein has adopted a similar binding site as that of the DNA damage-binding protein 2 (DDB2), which is known to form a complex with DDB1 to participate in UV-induced nucleotide excision repair [38], as well as in stimulating E2F1-activated transcription [39]. Experimental evidence exists that the V protein and DDB2 bind to DDB1 in a mutually exclusive manner [40]. Thus through sequestering DDB1 and inhibiting its association with DDB2, V proteins are expected to disrupt the normal function of the UV-DDB complex in DNA repair and cell cycle regulation, which are associated with the viral pathogenesis [41]. Through analyzing the structural models of the V protein-DDB1 complex and the UV-DDB complex, we again found significant interface similarity with a PC-score of 0.546, and it is clear from Figure 4D that such mimicry is established from the viruses’ rapid mutation leading to the converged interface, rather than from divergent evolution.

The last example of viral mimicry involves the mechanism of viral entry into host cells. Nipah viruses employ their attachment glycoprotein G (NiV-G) for anchoring to the cell surface before initiating membrane fusion, specifically via binding of the glycoprotein G to ephrin-B2 [42,43], which is a transmembrane ligand for the ephrin B class of receptor tyrosine kinases. Comparison of the NiV-G-ephrin-B2 complex and the cognate ephrin-B2-ephrin-B4 receptor complex reveals striking similarity in their structures [44,45], preserving key interactions at the G-H binding loop in both cases [46]. Although experimental evidence for the viral protein’s competitive binding to ephrin-B2 with the target protein’s cognate receptor remains to be established, the observed interface structural similarity has already spurred propositions of therapeutic schemes that target the anchor site of the viral protein while avoiding disrupting the endogenous ephrin receptor interactions [46]. Unsurprisingly, this interface mimicry is also captured by our method with a high PC-score of 0.430, demonstrating the power of our method in identifying shared patterns correlated with biological significance.

We also performed the same analysis using the two existing methods. While the non-sequential version of Ialign also detected significant similarity in all three cases, I2I-SiteEngine assigns scores that do not quantify these mimicked interfaces as sufficiently similar (Additional file 1: Table S6). Based on these anecdotal analyses of protein-protein interfaces bearing biologically significance with limited overall structural similarity in the constituent proteins, Ialign and PCalign appear to be the recommended methods for detecting the interface similarity when all structural details are available.

## Discussion and conclusions

Characterizing, classifying and annotating protein-protein interactions are fundamental to understanding the structural or functional relationship between proteins, and to provide additional insights into what can be revealed by studying individual proteins alone. Central to protein-protein interactions from a structural point of view is protein-protein interfaces, which may be dissimilar for similar monomers, and similar for dissimilar monomers. Structural comparison of protein-protein interfaces is thus expected to aid in organizing information hidden in the protein-protein interaction network, and enable predictions for novel biological functions undisclosed by protein monomer structures.

This work presents PCalign, a method to quantitatively measure interface similarity for a given pair of protein-protein interfaces, taking into account the chemical and spatial patterns of residues lining the interfaces. It primarily uses a geometric hashing algorithm to identify the optimal superimposition of two sets of discontinuous fragments of interfacial residues while disregarding their connectivity. Based on the optimal superimposition, a normalized scoring function, PC-score, is calculated to reflect the extent to which the two sets of interfacial residues overlap with each other in terms of their physicochemical properties. A major contribution of this new method is that PCalign adopts a coarse-grained approach in representing interfaces, aligning interfaces and scoring the alignment, therefore it is able to accommodate input data across different resolutions. This is expected to gain advantage over existing methods in the next era of structural bioinformatics, given the rate at which large macromolecular complexes solved at nano-resolutions continue to populate the pool of structural data. Performance-wise, we demonstrated that our method is comparable to existing methods in terms of computational complexity, and superior in terms of finding optimal structural alignment especially between unrelated pairwise protein interfaces, enabling detection of significant structural similarities that are sometimes missed by existing methods.

As PCalign is aimed at capturing the overall degree of equivalence between protein-protein interfaces, a necessary limitation with such a design is the lack of sensitivity towards local structural motifs shared among interfaces that are globally dissimilar (‘globally’ here refers to the entire interface, rather than the entire monomer structure). In such situations the local signal becomes diluted out upon normalization against the average interface size. In this regard, caution needs to be taken when screening for small signature motifs embedded in large interfaces using our method that was designed for measuring global similarity among interfaces.

With this new tool for protein-protein interface comparison, we would now like to expand our investigation of structural properties of protein-protein interfaces by analyzing large macromolecular assemblies, such as viral capsids. This class of proteins presents unique structural and functional characteristics unseen in cellular protein complexes [47], and the wealth of information contained in their structural data may enrich our knowledge of protein-protein interfaces in general. We expect PCalign to be a handy tool in exploring some interesting questions pertaining to the higher-order organization of these assemblies.

### Availability of software

The program PCalign, which operates on Linux systems, is freely available to academic users at http://brooks.chem.lsa.umich.edu/index.php?page=software&subdir=articles/resources.

## Additional files

Additional file 1:
**Supporting information for the main manuscript, including Supplemental figures S1 – S6 and Supplemental tables 1 – 6.**

Additional file 2:
**VMD files for Figure** 4 **.**
