# Supplementary material for: PCalign: a method to quantify physicochemical similarity of protein-protein interfaces
Source: BMC Bioinformatics. 2015 Feb 1;16:33. doi: 10.1186/s12859-015-0471-x (PMC4339745; doi:10.1186/s12859-015-0471-x)
Supplement: Additional file 1: — Supporting information for the main manuscript, including Supplemental figures S1 – S6 and Supplemental tables 1 – 6. [file 12859_2015_471_MOESM1_ESM.docx]

**Supplemental text**:

**
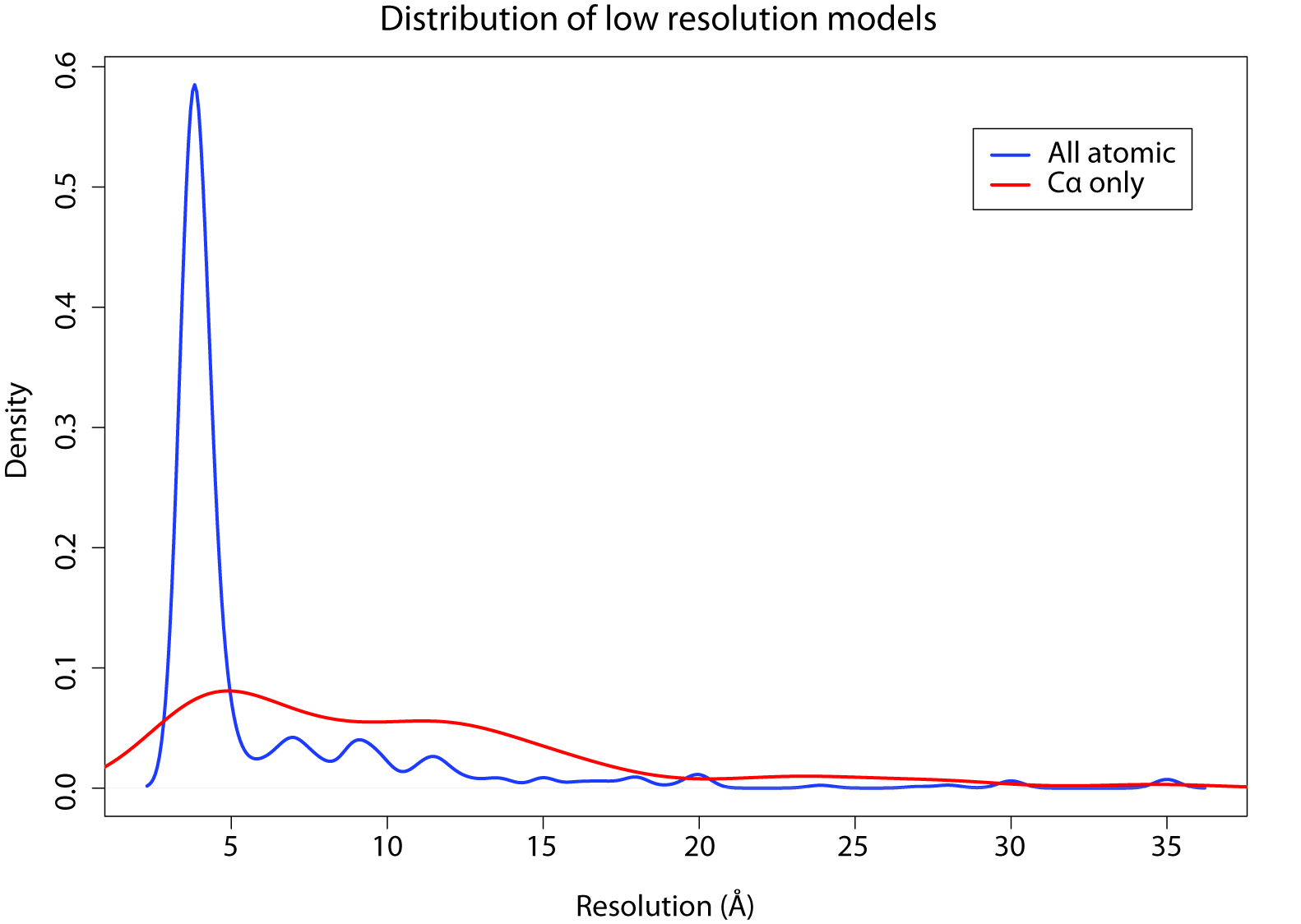
**

**Fig. S1.** Distribution of structural models in PDB with resolution lower than 3.5 Å. While models with all atomic details (shown in blue) are mostly clustered on the higher end of the resolution spectrum, structural models with coordinates of Cα atoms only, in comparison, are more likely to be populated on a wide range of resolutions, especially towards the lower end.

**Hierarchical definition of interfacial residues**:

To identify the optimal ξ value, we took a non-redundant set of 4248 protein dimers determined at atomic resolution, and selected all residues that had their Cα atom within 15Å of at least another Cα atom of a residue belonging to the binding partner. All pairs of residues, one from each chain, in each dimer then comprise our total set. We can compute the Matthews correlation coefficient (MCC) for each given ξ value,

$$\text{MCC}=\frac{\text{TP}\times\text{TN}-\text{FP}\times\text{FN}}{\sqrt{\left( \text{TP}+\text{FP} \right)\left( \text{TP}+\text{FN} \right)\left( \text{TN}+\text{FP} \right)(\text{TN}+\text{FN})}}$$

where true positives (TP) represent the number of residues that are in contact based on the heavy atoms within 4.5Å definition and “predicted” to be in contact based on the residue-specific Cα-Cα distance cutoff criterion with a given ξ value. False positives (FP) represent the number of residues that are not in contact based on the side chain definition but “predicted” to be in contact based on the backbone criterion. True negatives (TN) represent the number of residues that are not in contact based on the side chain criterion and also “predicted” to be not in contact based on the backbone criterion. False negatives (FN) represent the number of residues that are not in contact based on the side chain criterion but are “predicted” to be in contact based on the backbone criterion. Thus MCC gives a quantitative measure of how well the two definitions of contacting residues match for a given ξ value. We varied the ξ value, which stands for the fraction of the standard deviation to be added to the mean value, from -1.0 to 2.0, and chose the ξ value that maximizes the MCC with the side chain distance criterion. In addition, we also tested a range of generic Cα-Cα distance cutoffs (from 6Å to 12Å) that are invariant for the residue type and computed their respective MCC with the side chain distance criterion, in order to compare with the performance of our amino acid type-specific criterion.

In the range of ξ values we calculated in determining a good type-specific Cα-Cα distance cutoff, we found a ξ value of 0.5 gave the highest MCC value of about 0.48 (Supplemental Figure 2 in blue). In comparison, the optimal general Cα-Cα distance cutoff that is invariant to residue type, shown to be 8 Å, only yielded a MCC value of about 0.42 (Supplemental Figure 2 in red). A potential explanation for the fairly low MCC value in our residue type-specific criterion could be that, the rich repertoire of side-chain rotamers at the interface region results in large fluctuations in their Cα-Cα distances that almost cancel out any significant differences resulting from different residue types. This can be inferred from the comparable magnitudes in the standard deviation values of Cα-Cα distances across various types of residue pairs and in the difference between their mean values (Supplemental Table 1a, 1b). Despite the small improvement, given that the residue-type-specific Cα-Cα distance cutoff criterion matches better with the heavy-atom based criterion, we have incorporated this criterion with a ξ value of 0.5 into our hierarchical definition of contacting residues.

**
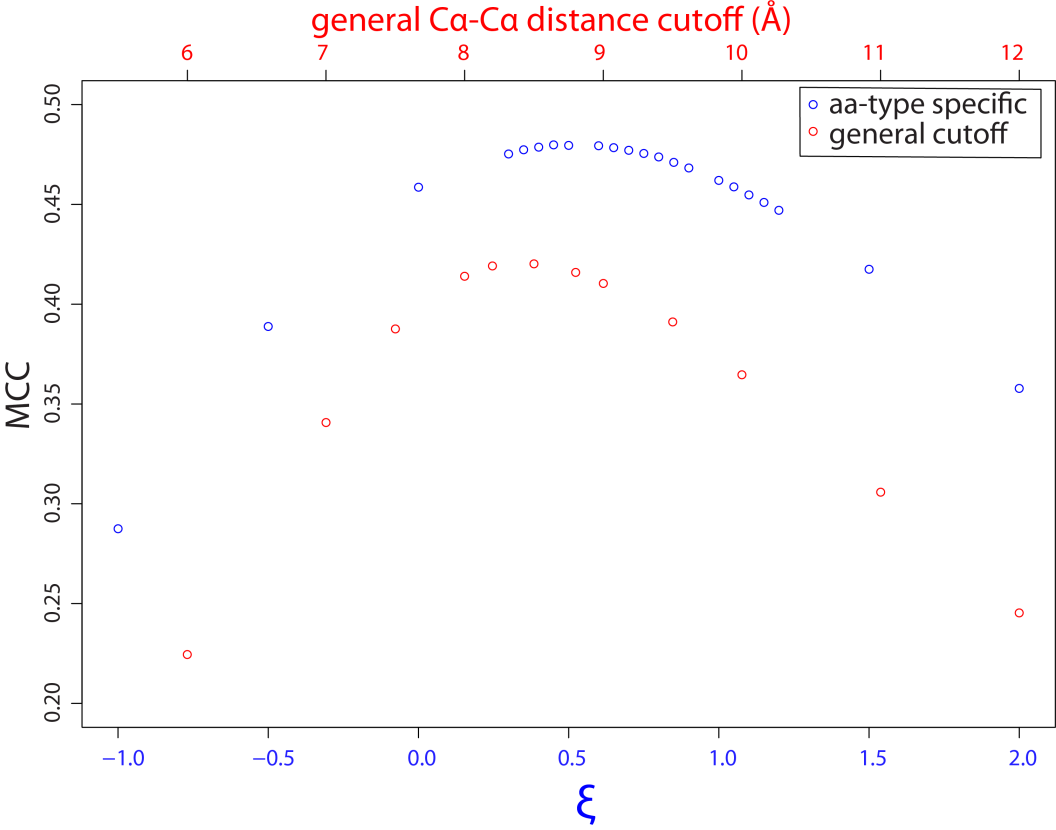
**

**Fig. S2.** Matthews Correlation Coefficient for the amino acid type-specific distance criterion and a general Cα-Cα distance cutoff criterion. The horizontal axis on top (red) gives the range of the distance cutoffs in Å we tested for the general Cα-Cα distance cutoff criterion, which resulted in a peak MCC value of 0.42 when the cutoff is chosen to be 8 Å (shown in red circles). The horizontal axis at the bottom (blue) shows the range of ξ values, which are the multiplicity factor of the standard deviation to be added to the mean value for each residue-residue type, and we obtained the highest MCC value of 0.48 when ξ = 0.5.

Supplemental Table 1a. Mean Cα-Cα distances for a given pair of contacting residues of specific types based on statistics in Protein Data Bank, where contacting residues are defined by having at least two heavy atoms, one from each residue, that are less than 4.5 Å apart.

|  | **A** | **R** | **N** | **D** | **C** | **E** | **Q** | **H** | **I** | **L** | **K** | **M** | **F** | **P** | **S** | **T** | **W** | **Y** | **V** |
| --- | --- | --- | --- | --- | --- | --- | --- | --- | --- | --- | --- | --- | --- | --- | --- | --- | --- | --- | --- |
| **A** | 5.53 | 7.99 | 6.42 | 6.24 | 6.03 | 6.76 | 6.97 | 6.91 | 6.74 | 6.97 | 6.96 | 7.14 | 7.59 | 6.10 | 5.70 | 6.27 | 7.89 | 7.68 | 6.36 |
| **R** | 7.99 | 10.31 | 8.80 | 9.32 | 8.02 | 9.87 | 9.21 | 9.29 | 8.84 | 8.73 | 9.88 | 8.95 | 9.32 | 8.40 | 8.41 | 8.57 | 9.30 | 9.75 | 8.40 |
| **N** | 6.42 | 8.80 | 7.31 | 7.46 | 6.65 | 7.95 | 7.79 | 8.12 | 7.33 | 7.61 | 8.37 | 8.02 | 8.23 | 6.83 | 6.82 | 7.01 | 8.78 | 8.71 | 7.09 |
| **D** | 6.24 | 9.32 | 7.46 | 7.53 | 6.64 | 8.06 | 8.06 | 8.34 | 7.32 | 7.27 | 8.77 | 7.66 | 8.00 | 6.73 | 6.68 | 6.81 | 8.72 | 8.98 | 6.87 |
| **C** | 6.03 | 8.02 | 6.65 | 6.64 | 5.93 | 7.09 | 7.69 | 7.97 | 7.27 | 7.43 | 7.58 | 7.59 | 8.08 | 6.64 | 6.37 | 6.54 | 8.48 | 7.99 | 6.74 |
| **E** | 6.76 | 9.87 | 7.95 | 8.06 | 7.09 | 8.63 | 8.49 | 8.90 | 7.61 | 7.86 | 9.40 | 8.07 | 8.39 | 7.40 | 7.36 | 7.47 | 9.11 | 9.31 | 7.35 |
| **Q** | 6.97 | 9.21 | 7.79 | 8.06 | 7.69 | 8.49 | 8.74 | 8.66 | 7.88 | 8.13 | 8.75 | 8.28 | 8.65 | 7.49 | 7.35 | 7.60 | 8.92 | 9.25 | 7.60 |
| **H** | 6.91 | 9.29 | 8.12 | 8.34 | 7.97 | 8.90 | 8.66 | 8.56 | 8.00 | 8.26 | 8.74 | 8.38 | 8.70 | 7.31 | 7.50 | 7.70 | 9.32 | 9.15 | 7.67 |
| **I** | 6.74 | 8.84 | 7.33 | 7.32 | 7.27 | 7.61 | 7.88 | 8.00 | 7.93 | 8.14 | 7.91 | 8.31 | 8.61 | 7.27 | 6.82 | 7.41 | 9.23 | 8.58 | 7.53 |
| **L** | 6.97 | 8.73 | 7.61 | 7.27 | 7.43 | 7.86 | 8.13 | 8.26 | 8.14 | 8.23 | 7.99 | 8.38 | 8.77 | 7.49 | 7.16 | 7.61 | 9.23 | 8.76 | 7.77 |
| **K** | 6.96 | 9.88 | 8.37 | 8.77 | 7.58 | 9.40 | 8.75 | 8.74 | 7.91 | 7.99 | 9.54 | 8.44 | 8.47 | 7.77 | 7.97 | 8.02 | 8.84 | 9.30 | 7.54 |
| **M** | 7.14 | 8.95 | 8.02 | 7.66 | 7.59 | 8.07 | 8.28 | 8.38 | 8.31 | 8.38 | 8.44 | 8.49 | 8.89 | 7.71 | 7.13 | 7.70 | 9.39 | 8.84 | 7.94 |
| **F** | 7.59 | 9.32 | 8.23 | 8.00 | 8.08 | 8.39 | 8.65 | 8.70 | 8.61 | 8.77 | 8.47 | 8.89 | 9.21 | 7.72 | 7.58 | 8.19 | 9.97 | 9.29 | 8.45 |
| **P** | 6.10 | 8.40 | 6.83 | 6.73 | 6.64 | 7.40 | 7.49 | 7.31 | 7.27 | 7.49 | 7.77 | 7.71 | 7.72 | 6.71 | 6.42 | 6.85 | 8.10 | 8.30 | 6.99 |
| **S** | 5.70 | 8.41 | 6.82 | 6.68 | 6.37 | 7.36 | 7.35 | 7.50 | 6.82 | 7.16 | 7.97 | 7.13 | 7.58 | 6.42 | 6.15 | 6.41 | 8.13 | 8.14 | 6.60 |
| **T** | 6.27 | 8.57 | 7.01 | 6.81 | 6.54 | 7.47 | 7.60 | 7.70 | 7.41 | 7.61 | 8.02 | 7.70 | 8.19 | 6.85 | 6.41 | 6.71 | 8.43 | 8.47 | 6.93 |
| **W** | 7.89 | 9.30 | 8.78 | 8.72 | 8.48 | 9.11 | 8.92 | 9.32 | 9.23 | 9.23 | 8.84 | 9.39 | 9.97 | 8.10 | 8.13 | 8.43 | 9.96 | 9.90 | 8.80 |
| **Y** | 7.68 | 9.75 | 8.71 | 8.98 | 7.99 | 9.31 | 9.25 | 9.15 | 8.58 | 8.76 | 9.30 | 8.84 | 9.29 | 8.30 | 8.14 | 8.47 | 9.90 | 9.37 | 8.40 |
| **V** | 6.36 | 8.40 | 7.09 | 6.87 | 6.74 | 7.35 | 7.60 | 7.67 | 7.53 | 7.77 | 7.54 | 7.94 | 8.45 | 6.99 | 6.60 | 6.93 | 8.80 | 8.40 | 7.11 |

Supplemental Table 1b. Standard deviation of Cα-Cα distances for a given pair of contacting residues of specific types based on statistics in Protein Data Bank, where contacting residues are defined by having at least two heavy atoms, one from each residue, that are less than 4.5 Å apart.

|  | **A** | **R** | **N** | **D** | **C** | **E** | **Q** | **H** | **I** | **L** | **K** | **M** | **F** | **P** | **S** | **T** | **W** | **Y** | **V** |
| --- | --- | --- | --- | --- | --- | --- | --- | --- | --- | --- | --- | --- | --- | --- | --- | --- | --- | --- | --- |
| **A** | 0.84 | 1.94 | 1.04 | 1.06 | 0.92 | 1.28 | 1.32 | 1.37 | 1.07 | 1.13 | 1.65 | 1.43 | 1.48 | 0.81 | 0.87 | 0.88 | 1.78 | 1.73 | 0.84 |
| **R** | 1.94 | 2.70 | 2.14 | 2.02 | 1.94 | 2.23 | 2.21 | 2.19 | 2.03 | 1.95 | 2.52 | 2.08 | 2.14 | 1.95 | 2.02 | 2.02 | 2.24 | 2.47 | 1.92 |
| **N** | 1.04 | 2.14 | 1.45 | 1.44 | 1.31 | 1.58 | 1.70 | 1.49 | 1.37 | 1.43 | 1.96 | 1.62 | 1.79 | 1.18 | 1.25 | 1.32 | 1.85 | 2.00 | 1.18 |
| **D** | 1.06 | 2.02 | 1.44 | 1.67 | 1.24 | 1.80 | 1.65 | 1.61 | 1.27 | 1.42 | 1.89 | 1.52 | 1.73 | 1.19 | 1.08 | 1.22 | 1.95 | 1.90 | 1.12 |
| **C** | 0.92 | 1.94 | 1.31 | 1.24 | 1.19 | 1.44 | 1.42 | 1.35 | 1.30 | 1.18 | 1.81 | 1.48 | 2.05 | 1.04 | 1.04 | 1.11 | 2.10 | 1.89 | 1.19 |
| **E** | 1.28 | 2.23 | 1.58 | 1.80 | 1.44 | 2.10 | 1.79 | 1.85 | 1.48 | 1.41 | 2.09 | 1.59 | 1.77 | 1.32 | 1.38 | 1.40 | 1.97 | 2.13 | 1.28 |
| **Q** | 1.32 | 2.21 | 1.70 | 1.65 | 1.42 | 1.79 | 1.95 | 1.87 | 1.51 | 1.50 | 2.10 | 1.73 | 1.87 | 1.41 | 1.44 | 1.41 | 1.99 | 2.24 | 1.40 |
| **H** | 1.37 | 2.19 | 1.49 | 1.61 | 1.35 | 1.85 | 1.87 | 2.09 | 1.63 | 1.49 | 2.04 | 1.81 | 1.75 | 1.40 | 1.47 | 1.50 | 2.14 | 2.19 | 1.42 |
| **I** | 1.07 | 2.03 | 1.37 | 1.27 | 1.30 | 1.48 | 1.51 | 1.63 | 1.47 | 1.40 | 1.67 | 1.59 | 1.70 | 1.14 | 1.19 | 1.29 | 1.90 | 1.88 | 1.28 |
| **L** | 1.13 | 1.95 | 1.43 | 1.42 | 1.18 | 1.41 | 1.50 | 1.49 | 1.40 | 1.51 | 1.63 | 1.62 | 1.83 | 1.24 | 1.28 | 1.25 | 1.96 | 1.81 | 1.23 |
| **K** | 1.65 | 2.52 | 1.96 | 1.89 | 1.81 | 2.09 | 2.10 | 2.04 | 1.67 | 1.63 | 2.95 | 1.92 | 1.89 | 1.77 | 1.87 | 1.94 | 2.10 | 2.32 | 1.57 |
| **M** | 1.43 | 2.08 | 1.62 | 1.52 | 1.48 | 1.59 | 1.73 | 1.81 | 1.59 | 1.62 | 1.92 | 1.92 | 1.93 | 1.45 | 1.59 | 1.56 | 2.15 | 2.06 | 1.57 |
| **F** | 1.48 | 2.14 | 1.79 | 1.73 | 2.05 | 1.77 | 1.87 | 1.75 | 1.70 | 1.83 | 1.89 | 1.93 | 2.16 | 1.56 | 1.55 | 1.74 | 2.45 | 2.16 | 1.66 |
| **P** | 0.81 | 1.95 | 1.18 | 1.19 | 1.04 | 1.32 | 1.41 | 1.40 | 1.14 | 1.24 | 1.77 | 1.45 | 1.56 | 1.17 | 0.98 | 1.01 | 1.61 | 1.77 | 0.99 |
| **S** | 0.87 | 2.02 | 1.25 | 1.08 | 1.04 | 1.38 | 1.44 | 1.47 | 1.19 | 1.28 | 1.87 | 1.59 | 1.55 | 0.98 | 1.10 | 1.10 | 1.81 | 1.91 | 1.04 |
| **T** | 0.88 | 2.02 | 1.32 | 1.22 | 1.11 | 1.40 | 1.41 | 1.50 | 1.29 | 1.25 | 1.94 | 1.56 | 1.74 | 1.01 | 1.10 | 1.25 | 1.92 | 1.93 | 1.10 |
| **W** | 1.78 | 2.24 | 1.85 | 1.95 | 2.10 | 1.97 | 1.99 | 2.14 | 1.90 | 1.96 | 2.10 | 2.15 | 2.45 | 1.61 | 1.81 | 1.92 | 2.69 | 2.34 | 1.87 |
| **Y** | 1.73 | 2.47 | 2.00 | 1.90 | 1.89 | 2.13 | 2.24 | 2.19 | 1.88 | 1.81 | 2.32 | 2.06 | 2.16 | 1.77 | 1.91 | 1.93 | 2.34 | 2.67 | 1.84 |
| **V** | 0.84 | 1.92 | 1.18 | 1.12 | 1.19 | 1.28 | 1.40 | 1.42 | 1.28 | 1.23 | 1.57 | 1.57 | 1.66 | 0.99 | 1.04 | 1.10 | 1.87 | 1.84 | 1.15 |

**Geometric hashing**:

Geometric hashing is divided into two phases; the construction phase and the voting phase. The construction phase constructs a lookup table for each structure, and is computed only once. The voting phase retrieves the two tables corresponding to the two structures being compared and finds the best transformations to apply to one of the interfaces to be overlaid with the other.

In the construction phase, a lookup table is built for an interface, which describes the projected coordinates of each point (residue) based on different reference bases. Each orthogonal basis ($x$,$y$,$z$) is defined by an ordered triplet of points (residues *i*, *j*, *k*, which are interfacial residues that occur consecutively in their sequence order) in the following manner,

$\boldsymbol{x}=\boldsymbol{v}_{\boldsymbol{i}}-\boldsymbol{v}_{\boldsymbol{j}}$**,**

$\boldsymbol{y}^{'}=\boldsymbol{v}_{\boldsymbol{k}}-\boldsymbol{v}_{\boldsymbol{j}}$**,**

$\boldsymbol{z}=\boldsymbol{x}\times\boldsymbol{y}^{'}$**,**

$\boldsymbol{y}=\boldsymbol{z}\times\boldsymbol{x}$**.**

where $v_{i}, v_{j}$ and $v_{k}$ are the coordinates of residues *i*, *j*, *k* respectively. The $\times$ operation refers to the cross product. Each point is then projected onto this particular reference basis to obtain its new coordinates, which are then discretized based on a given grid size (we used 4 Å in our case). The new, discretized coordinates of the point (a feature) is then recorded as a hash key pointing to the basis that leads to this specific transformation. All possible ordered triplets are computed and registered into the lookup table based on their transformed coordinates (the features).

The voting procedure browses the two lookup tables that have been pre-computed and finds pairs of orthogonal bases that generate a sufficiently large number of features that match. As we look up each hash key (feature) in the first table, we find the matching key in the second table, and give one vote to all the pairs of orthogonal bases pointed by this feature in the two tables. Consequently pairs of orthogonal bases that receive a high vote count correspond to bases to re-orient the two structures so that they have many points that overlap. These bases are then used to compute candidate initial alignments. Our extensive tests show that this approach yields the final optimal score with sufficient sampling (Supplemental Table 2).

Supplemental Table 2. Percentage of correctly mapped interfacial residues by PCalign in quasi-equivalent protein-protein interfaces within the same capsid. In quasi-equivalent inter-subunit interfaces within a capsid, structurally equivalent residues have an exact one-to-one correspondence due to the monomers being sequence-wise identical. It is evident from these numbers that our sampling is sufficient in correctly assigning equivalence to pairs of residues for which the correspondence is known. There are, however, a few cases where not all matched pairs have the same residue ID, and they mostly occur in the comparison between A1B5 and C1C6, and between A1A2 and B1C6. These comparisons are between a “bent” interface on a five-fold symmetry axis and a “flat” interface on a six-fold symmetry axis, which are therefore less equivalent, as reflected by the lower PC-scores in the brackets. In these cases, the “mismatches” identified by the program are the consequence of maximizing the overlap of physicochemical patterns between less equivalent interfaces, rather than having resulted from inefficient sampling.

| Virus name | PDB ID | A1B1: B1C1 | A1C1: B1C1 | A1B1: A1C1 | A1B5: C1-C6 | A1A2: B1C2 | A1A2: B1C6 | B1C2: B1C6 |
| --- | --- | --- | --- | --- | --- | --- | --- | --- |
| TNV | 1c8n | 100% | 100% | 100% | 100% | 100% | 100% | 97% (0.483) |
| SMV | 1smv | 100% | 100% | 100% | 100% | 100% | 100% | 100% |
| SBMV | 4sbv | 100% | 100% | 100% | 97% (0.722) | 100% | 100% | 100% |
| RYMV | 1f2n | 100% | 100% | 100% | 100% | 100% | 100% | 100% |
| BBV | 2bbv | 100% | 100% | 100% | 91% (0.595) | 100% | 95% (0.597) | 100% |
| NOV | 1nov | 100% | 100% | 100% | 96% (0.558) | 100% | 100% | 85% (0.495) |
| PAV | 1f8v | 100% | 100% | 100% | 88% (0.433) | 100% | 93% (0.400) | 100% |
| TBSV | 2tbv | 100% | 100% | 100% | 100% | 100% | 100% | 100% |
| NV | 1ihm | 100% | 100% | 100% | 100% | 100% | 98% (0.737) | 100% |
| CCMV | 1cwp | 100% | 100% | 100% | 100% | 100% | 96% (0.545) | 100% |
| CMV | 1f15 | 100% | 100% | 100% | 100% | - | - | 100% |
| TYMV | 1auy | 97% (0.722) | 100% | 100% | 100% | 100% | 92% (0.715) | 100% |
| DYMV | 1ddl | 100% | 94% (0.620) | 100% | 100% | 100% | 100% | 100% |
| PhMV | 1qjz | 100% | 100% | 100% | 100% | 94% (0.695) | 97% (0.569) | 97% (0.698) |
|  |  |  |  |  |  | B1B2 : A1C6 | B1B2 : C1A2 | A1C6 : C1A2 |
| GA | 1gav | 100% | 94% (0.748) | 100% | 100% | 100% | 100% | 100% |
| FR | 1frs | 100% | 100% | 100% | 100% | 100% | - | 91% (0.700) |
| MS2 | 2ms2 | 100% | 100% | 100% | 100% | 100% | - | 95% (0.693) |
| QB | 1qbe | 94% (0.772) | 92% (0.680) | 100% | 100% | 100% | 100% | 87% (0.755) |

**Hungarian algorithm**:

The Hungarian algorithm is used for finding maximal weight matching in bipartite graphs ([Kuhn, 2005](#_ENREF_3)). Briefly, the algorithm starts with some initial state, and iteratively improves the objective function until it is optimized.

Key definitions included in this algorithm are as follows:

A bipartite graph, *G*(*V*,*E*), consists of two sets of notes, *X* and *Y*, that satisfy *V*= *X* $\cup$ *Y* and *X* $\cap$ *Y* = $\emptyset$, and the set of edges *E* $\subseteq$ *X* $\times$ *Y*. The weights of the edges are given by *w*(*x*,*y*) for *x* $\in$ *X*, *y* $\in$ *Y*. A matching is a subset of edges, *M* $\subseteq$ *E*, such that $\forall$*v* $\in$ *V*, at most one edge in *M* is incident upon *v*. The neighborhood of *v*, *N*(*v*), is all vertices that share an edge with *v*, and the neighborhood of a set of vertices *S*, *N*(*S*), is all vertices that share an edge with a vertix in *S*. The vertex labelling function is defined by *l*: *V*$\to$ *R*, which is feasible if *l*(*x*) + *l*(*y*) $\geq$ *w*(*x*,*y*) $\forall$ *x* $\in$ *X*, *y* $\in$ *Y*. For *G_l_*(*V*,*E_l_*), if *E_l_* satisfies *E_l_* = {(*x*,*y*): *l*(*x*)+*l*(*y*) = *w*(*x*,*y*)}, then *G_l_*(*V*,*E_l_*) is an equality subgraph.

Based on Kuhn-Munkres Theorem, if *l* is feasible and *M* is a perfect matching in *E_l_*, then *M* is a maximum weight matching, which is what the algorithm aims to find.

The Hungarian algorithm works as such:

Initialization: $\forall$*y*$\in$*Y*, *l*(*y*)=0; $\forall$*x*$\in$*X*, *l*(*x*) = $\max_{y\in Y} \{w(x,y)\}$. Set *M*=$\emptyset$.

Iteration: while *M* is not perfect,

1. Choose an unmatched vertex *u*$\boldsymbol{\in}$*X*, and set *S*={*u*},*T*=$\boldsymbol{\emptyset}$.
2. If the *N_l_*(*S*) = *T*, define a slack variable, $\boldsymbol{\delta}$=$\min_{\boldsymbol{u\in S, y\in Y\backslash T}} \boldsymbol{(l}\left( \boldsymbol{x} \right)\boldsymbol{+l}\left( \boldsymbol{y} \right)\boldsymbol{-w}\left( \boldsymbol{x,y} \right)\boldsymbol{)}$, and update labelling as such:

*l*′(*v*) = $\left\{ \begin{aligned} l\left( v \right)-\delta, v\epsilon S \\ l\left( v \right)+\delta\text{,} v\epsilon T \\ l\left( v \right), \text{otherwise} \end{aligned} \right.$

1. If the *N_l_*(*S)* ≠ *T*, pick *y* $\boldsymbol{\in}$ *N_l_*(*S*) $\boldsymbol{-}$ *T*,

If y is free, augment M and go back to while loop.

If y is matched to some vertex *z*, extend the alternating tree by setting *S* = *S* $\cup\{z\}$, *T* = *T* $\cup$ {*y*}. Go back to 2.

When the iteration stops, *M* is perfect, and it is the maximum weight matching we look for. In the search for structurally equivalent residues across different interfaces, we define the weight function, *w*(*x*,*y*), by the equivalence-score (Equation 2) in the Methods section.

**Correcting for length-dependency of raw PC-score**:

Earlier studies have reported the dependency of raw alignment scores on the sizes of interfaces being compared ([Gao and Skolnick, 2010](#_ENREF_2)). We therefore applied a similar approach to make the scoring function independent of the interface size, by considering the mean PC-score_raw_ of all random pairs whose interface sizes are $\pm$5% of a given size. When performed on a non-redundant set of 1797 interfaces, we observed that the raw score showed exponential dependence on the interface size, as shown in Supplemental Figure 3.


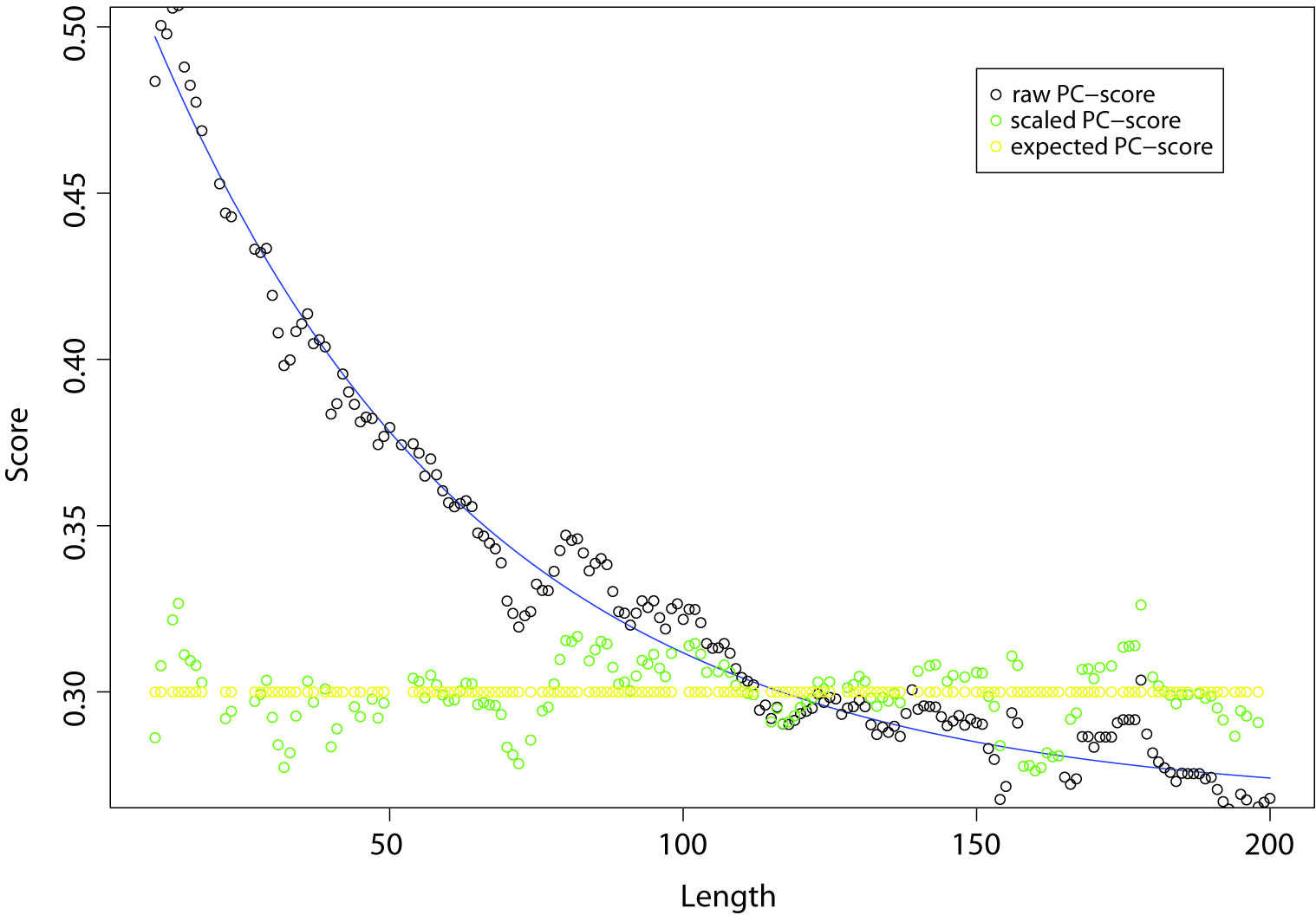


**Fig. S3.** The mean of PC-score_raw_ across random, unrelated pairs whose interface sizes are between 95% and 105% of a given interface size. The length-corrected PC-score based on Equation 5 of the main text, derived from the curve fitting here, becomes 0.3 for randomly chosen interface pairs regardless of their size.

**Statistical significance of PC-scores**:

To derive the p-value of any PC-score, we generated the distribution of PC-scores for comparing 1,613,706 pairs of interfaces, using the same dataset as in Fig. S3. Based on the distribution of PC-scores for random alignments, shown in Fig. S4, we can estimate the p-value of a given PC-score *s* empirically by obtaining the area under the curve to the right of *s*. A few representative p-values and their corresponding PC-scores are provided in Supplemental Table 3.


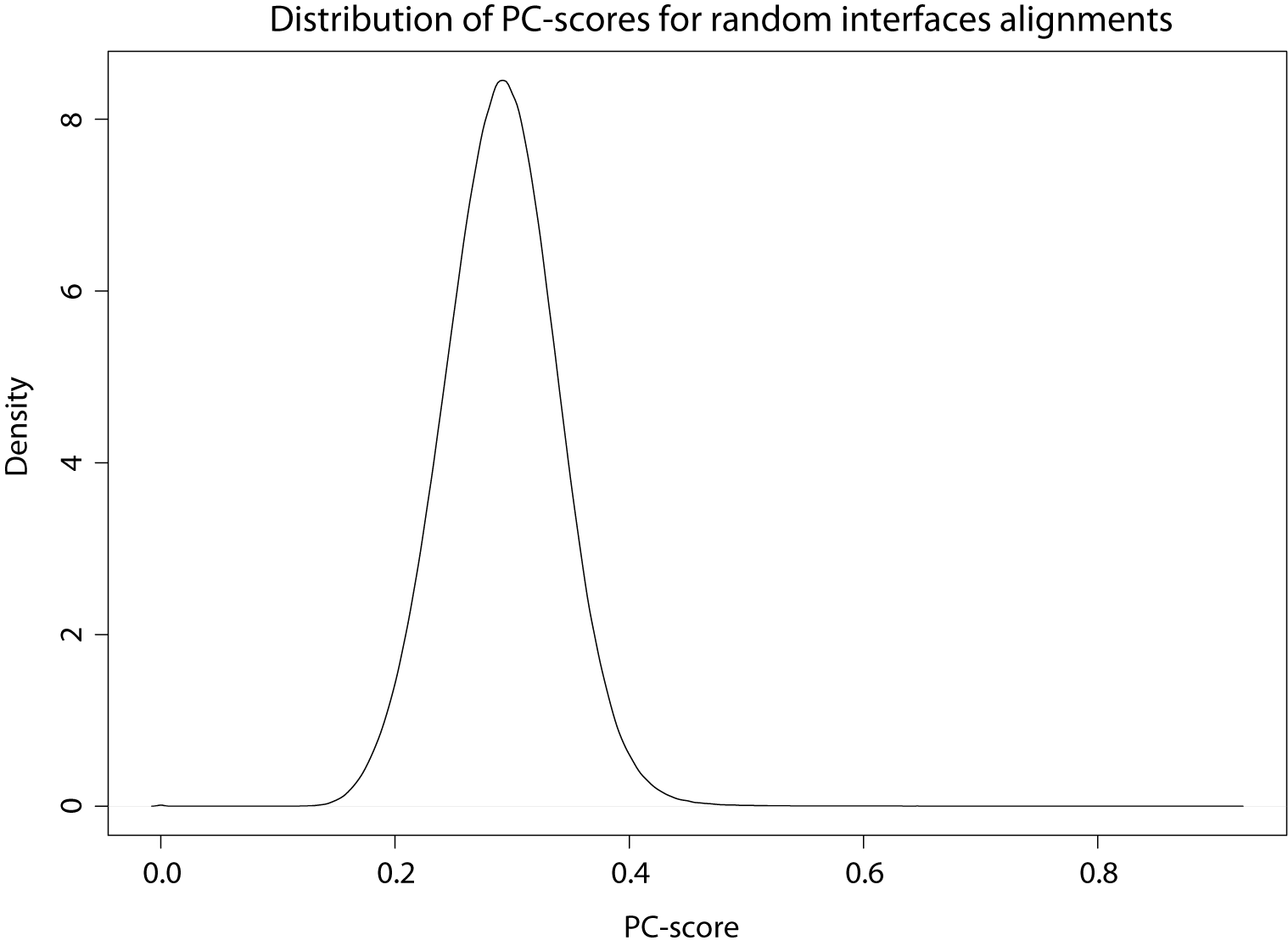


Fig. S4. The distribution of PC-scores, obtained by tabulating the all-against-all pairwise interface comparison for a non-redundant set of 1797 interfaces.

Supplemental Table 3. Statistical significance of PC-scores.

| p-value | 0.05 | 0.01 | 0.005 | 0.001 | 0.0005 | 0.0001 | 0.00001 |
| --- | --- | --- | --- | --- | --- | --- | --- |
| PC-score | 0.370 | 0.407 | 0.424 | 0.483 | 0.541 | 0.699 | 0.849 |

Supplemental Table 4. The interface equivalence measured by Q-score and PC-score for different T=3 viruses. The “/” separates the Q-score reported in ([Damodaran, et al., 2002](#_ENREF_1)) and the PC-score computed using our method. The two scoring functions match well with an overall correlation coefficient of 0.93, and show better agreement in some viruses than in others (last column).

| **Virus name*** | **PDB ID** | **A1B1:B1C1** | **A1C1:B1C1** | **A1B1:A1C1** | **A1B5:C1C6** | **A1A2:B1C2** | **A1A2:B1C6** | **B1C2:B1C6** | **c.c. within virus** |
| --- | --- | --- | --- | --- | --- | --- | --- | --- | --- |
| **TNV** | 1c8n | 0.93/0.97 | 0.95/0.96 | 0.98/0.97 | 0.47/0.53 | 0.81/0.84 | 0.30/0.58 | 0.29/0.52 | 0.97 |
| **SMV** | 1smv | 0.97/0.93 | 0.97/0.94 | 0.97/0.97 | 0.38/0.57 | 0.76/0.78 | 0.37/0.58 | 0.38/0.55 | 0.99 |
| **SBMV** | 4sbv | 0.92/0.93 | 0.90/0.94 | 0.93/0.95 | 0.36/0.52 | 0.74/0.80 | 0.28/0.52 | 0.26/0.53 | 0.99 |
| **RYMV** | 1f2n | 0.70/0.75 | 0.73/0.76 | 0.94/0.95 | 0.15/0.33 | 0.93/0.92 | 0.53/0.47 | 0.52/0.49 | 0.96 |
| **BBV** | 2bbv | 0.90/0.88 | 0.93/0.94 | 0.90/0.90 | 0.33/0.42 | 0.88/0.90 | 0.54/0.45 | 0.53/0.47 | 0.97 |
| **NOV** | 1nov | 0.82/0.93 | 0.91/0.93 | 0.86/0.96 | 0.40/0.41 | 0.87/0.86 | 0.41/0.47 | 0.44/0.47 | 0.98 |
| **PAV** | 1f8v | 0.70/0.80 | 0.78/0.86 | 0.72/0.79 | 0.29/0.37 | 0.74/0.81 | 0.33/0.41 | 0.42/0.48 | 1.00 |
| **TBSV** | 2tbv | 0.87/0.84 | 0.90/0.83 | 0.85/0.91 | 0.77/0.77 | 0.86/0.83 | 0.44/0.55 | 0.42/0.53 | 0.97 |
| **NV** | 1ihm | 0.77/0.79 | 0.74/0.82 | 0.84/0.87 | 0.96/0.93 | 0.63/0.70 | 0.69/0.70 | 0.58/0.66 | 0.97 |
| **CCMV** | 1cwp | 0.77/0.66 | 0.80/0.67 | 0.74/0.68 | 0.90/0.83 | 0.46/0.58 | 0.47/0.56 | 0.96/0.96 | 0.88 |
| **CMV** | 1f15 | 0.92/0.74 | 0.71/0.7 | 0.77/0.87 | 0.78/0.83 | - | - | 0.92/0.92 | 0.36 |
| **TYMV** | 1auy | 0.59/0.73 | 0.80/0.83 | 0.68/0.78 | 0.88/0.90 | 0.54/0.66 | 0.57/0.70 | 0.85/0.89 | 0.99 |
| **DYMV** | 1ddl | 0.79/0.81 | 0.56/0.65 | 0.71/0.76 | 0.93/0.92 | 0.72/0.82 | 0.78/0.79 | 0.90/0.89 | 0.97 |
| **PhMV** | 1qjz | 0.51/0.62 | 0.66/0.67 | 0.51/0.59 | 0.49/0.63 | 0.42/0.59 | 0.44/0.52 | 0.42/0.62 | 0.66 |
|  |  |  |  |  |  | **B1B2:A1C6** | **B1B2:C1A2** | **A1C6:C1A2** |  |
| **GA** | 1gav | 0.55/0.62 | 0.62/0.60 | 0.74/0.84 | 0.88/0.92 | 0.55/0.53 | 0.34/0.51 | 0.79/0.79 | 0.92 |
| **FR** | 1frs | 0.52/0.62 | 0.57/0.62 | 0.74/0.73 | 0.86/0.92 | 0.52/0.61 | - | 0.45/0.55 | 0.97 |
| **MS2** | 2ms2 | 0.39/0.56 | 0.52/0.56 | 0.71/0.78 | 0.92/0.94 | 0.50/0.57 | - | 0.45/0.64 | 0.95 |
| **QB** | 1qbe | 0.44/0.70 | 0.69/0.66 | 0.64/0.68 | 0.92/0.94 | 0.58/0.57 | 0.36/0.47 | 0.60/0.65 | 0.84 |

*Tobacco necrosis virus (TNV), Sesbania mosaic virus (SMV), Southern bean mosaic virus (SBMV), Rice yellow mottle virus (RYMV), Black beetle virus (BBV), Nodamura virus (NOV), Pariacoto virus (PAV), Tomato bushy stunt virus (TBSV), Norwalk virus (NV), Cowpea chlorotic mottle virus (CCMV), Cucumber mosaic virus (CMV), Turnip yellow mosaic virus (TYMV), Desmodium yellow mottle virus (DYMV), Physalis mottle virus (PhMV), Bacteriophage GA (GA), Bacteriophage FR (FR), Bacteriophage MS2 (MS2), Bacteriophage Q beta (QB).

The following case in Fig. S5 gives an example where PCalign outperforms Ialign in finding better structural alignment between two unrelated interfaces. For this pair of unrelated dimers, one being a chorismate mutase and the other being a hypothetical protein with unknown function, the monomers share an overall low structural similarity, measured by a low Template Modeling-score (TM-score) ([Zhang and Skolnick, 2004](#_ENREF_5)) of 0.29132. However, PCalign recognizes significant interface similarity that is missed by Ialign, by finding structural alignment that has higher coverage and lower RMSD.


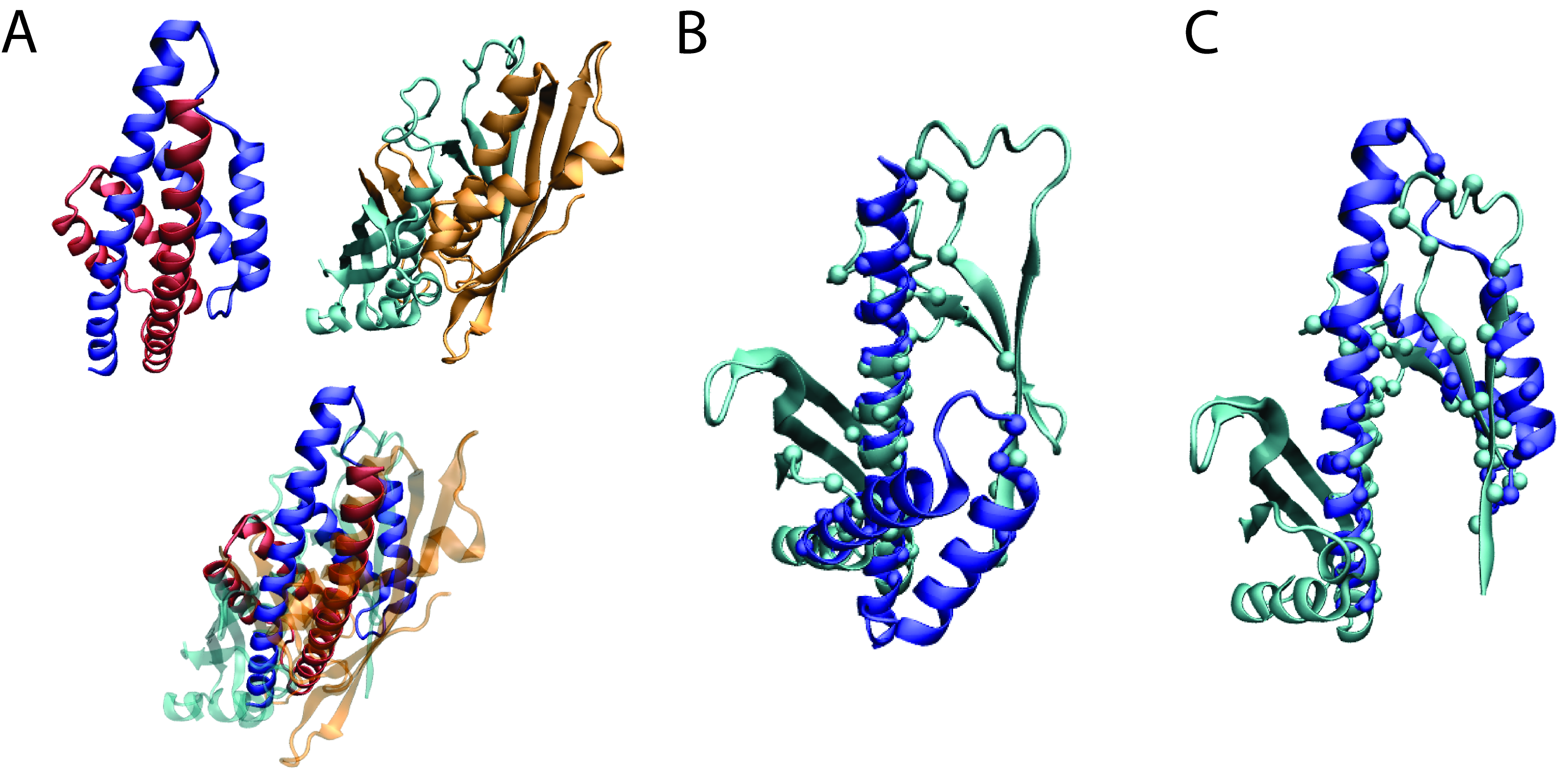


**Fig. S5**. An example of similar interfaces across unrelated dimers missed by existing methods but captured by PCalign. (A) The homodimer [PDB: 2D8D] in blue and red and the homodimer [PDB: 2D7V] in cyan and orange show no global structural similarity in their monomers, but both dimers formed intertwined interfaces in a fashion that resembles two hairpins clipped to each other. (B) Ialign aligns 74 residues out of 103 interfacial residues of the smaller interface with an RMSD of 3.87Å, and the IS-score of 0.220 has a p-value > 0.05 which makes the interface similarity insignificant. (C) PCalign aligns 93 out of 103 interfacial residues of the smaller interface with an RMSD of 3.47 Å, and the PC-score of 0.418 has a p-value of 0.007, which regards the interface similarity as significantly high. This is illustrated by the interface alignment showing just one binding site, given the interface is symmetric. Figures are generated by the VMD software.

Supplemental Table 5. Protein dimers grouped by their familiar annotation by SCOPPI. All dimers within the same group are less than 50% identical sequence-wise, and have the same dimerization states. The first alphabet and the first number of the SCOP classification describe the folded topology of the monomer structure, the third number representing its superfamily and the last its family. As shown in the table, the selected groups of protein dimers cover a diverse range of folds, and there are no “shared” monomers (in terms of their familial annotation) across different groups.

| **Grp** | **PDB ID** | **Family of chain 1** | **SCOP classification** | **Family of chain 2** | **SCOP classification** |
| --- | --- | --- | --- | --- | --- |
| 1 | 1all_AB | Phycocyanin-like phycobilisome proteins | a.1.1.3 | Phycocyanin-like phycobilisome proteins | a.1.1.3 |
|  | 1eyx_AB |  |  |  |  |
|  | 1ha7_AB |  |  |  |  |
| 2 | 3bro_AB | MarR-like transcriptional regulators | a.4.5.28 | MarR-like transcriptional regulators | a.4.5.28 |
|  | 1lj9_AB |  |  |  |  |
|  | 2a61_AB |  |  |  |  |
|  | 2hr3_AB |  |  |  |  |
|  | 2fxa_AB |  |  |  |  |
|  | 1s3j_AB |  |  |  |  |
|  | 1hsj_AB |  |  |  |  |
|  | 1z9c_AB |  |  |  |  |
|  | 1lnw_AB |  |  |  |  |
|  | 2frh_AB |  |  |  |  |
|  | 2eth_AB |  |  |  |  |
|  | 2fbk_AB |  |  |  |  |
| 3 | 1nek_AB | Succinate dehydrogenase/ fumarate reductase flavoprotein C-terminal domain | a.7.3.1 | 2Fe-2S ferredoxin domains from multidomain proteins | d.15.4.2 |
|  | 1e7p_AB |  |  |  |  |
|  | 1kfy_AB |  |  |  |  |
| 4 | 2f8n_DK | Nucleosome core histones | a.22.1.1 | Nucleosome core histones | a.22.1.1 |
|  | 2aro_AB |  |  |  |  |
|  | 2fj7_AB |  |  |  |  |
| 5 | 2yw7_AE | Ferritin | a.25.1.1 | Ferritin | a.25.1.1 |
|  | 1ji4_AD |  |  |  |  |
|  | 2bkc_AC |  |  |  |  |
|  | 2ux1_AC |  |  |  |  |
|  | 1eum_AF |  |  |  |  |
|  | 1sq3_AB |  |  |  |  |
|  | 1vlg_AF |  |  |  |  |
|  | 1n1q_AB |  |  |  |  |
|  | 1nf4_AB |  |  |  |  |
|  | 2fjc_AD |  |  |  |  |
|  | 2htn_AB |  |  |  |  |
|  | 1mfr_AG |  |  |  |  |
|  | 1jre_AB |  |  |  |  |
|  | 1zuj_AB |  |  |  |  |
|  | 1z6o_AM |  |  |  |  |
|  | 1tk6_AB |  |  |  |  |
| 6 | 1fg9_AC | Interferons/interleukin-10 (IL-10) | a.26.1.3 | Fibronectin type III | b.1.2.1 |
|  | 1j7v_LR |  |  |  |  |
|  | 1lqs_LR |  |  |  |  |
| 7 | 2cx9_AB | Medium chain acyl-CoA dehydrogenase-like, C-terminal domain | a.29.3.1 | Medium chain acyl-CoA dehydrogenase, NM (N-terminal and middle) domains) | e.6.1.1 |
|  | 2reh_AB |  |  |  |  |
|  | 1t9g_AB |  |  |  |  |
|  | 1ivh_AB |  |  |  |  |
|  | 1jqi_AB |  |  |  |  |
|  | 1rx0_AC |  |  |  |  |
| 8 | 2gzu_AB | SinR domain-like | a.35.1.3 | SinR domain-like | a.35.1.3 |
|  | 1zzc_AB |  |  |  |  |
|  | 1y7y_AB |  |  |  |  |
|  | 2b5a_AB |  |  |  |  |
|  | 2ofy_AB |  |  |  |  |
| 9 | 2mys_AC | Calmodulin-like | a.39.1.5 | Motor proteins | c.37.1.9 |
|  | 1w7i_AB |  |  |  |  |
|  | 1br1_AB |  |  |  |  |
|  | 1l2o_AC |  |  |  |  |
| 10 | 1ea4_AB | CopG-like | a.43.1.3 | CopG-like | a.43.1.3 |
|  | 1x93_AB |  |  |  |  |
|  | 1p94_AB |  |  |  |  |
| 11 | 1v2a_AB | Glutathione S-transferase (GST), C-terminal domain | a.45.1.1 | Glutathione S-transferase (GST), N-terminal domain | c.47.1.5 |
|  | 1tu7_AB |  |  |  |  |
|  | 1pn9_AB |  |  |  |  |
|  | 1tw9_AB |  |  |  |  |
|  | 2aaw_AC |  |  |  |  |
|  | 1y6e_AB |  |  |  |  |
|  | 2c8u_AB |  |  |  |  |
|  | 1pl2_AB |  |  |  |  |
|  | 1zgn_AB |  |  |  |  |
|  | 1m0u_AB |  |  |  |  |
|  | 2ab6_AB |  |  |  |  |
|  | 1gul_AB |  |  |  |  |
|  | 1gwc_BC |  |  |  |  |
|  | 1oyj_AB |  |  |  |  |
|  | 1k0c_AB |  |  |  |  |
|  | 1v40_AD |  |  |  |  |
|  | 1axd_AB |  |  |  |  |
|  | 2pmt_AB |  |  |  |  |
|  | 1n2a_AB |  |  |  |  |
|  | 1ljr_AB |  |  |  |  |
| 12 | 2elc_AD | Nucleoside phosphorylase/phosphoribosyltransferase N-terminal domain | a.46.2.1 | (Nucleoside phosphorylase/phosphoribosyltransferase N-terminal domain | a.46.2.1 |
|  | 1kgz_AB |  |  |  |  |
|  | 2gvq_AD |  |  |  |  |
|  | 1brw_AB |  |  |  |  |
|  | 1azy_AB |  |  |  |  |
| 13 | 1p51_AB | Prokaryotic DNA-bending protein) | a.55.1.1 | Prokaryotic DNA-bending protein | a.55.1.1 |
|  | 1b8z_AB |  |  |  |  |
|  | 1exe_AB |  |  |  |  |
|  | 2o97_AB |  |  |  |  |
|  | 1ihf_AB |  |  |  |  |
| 14 | 2a1j_AB | Hef domain-like | a.60.2.5 | Hef domain-like | a.60.2.5 |
|  | 1x2i_AB |  |  |  |  |
|  | 2aq0_AB |  |  |  |  |
|  | 1z00_AB |  |  |  |  |
| 15 | 1f5q_AB | Cyclin | a.74.1.1 | Protein kinases, catalytic subunit | d.144.1.7 |
|  | 1g3n_AC |  |  |  |  |
|  | 1xo2_AB |  |  |  |  |
|  | 2f2c_AB |  |  |  |  |
|  | 1fvv_AB |  |  |  |  |
|  | 1w98_AB |  |  |  |  |
| 16 | 2nz8_AB | DBL homology domain (DH-domain) | a.87.1.1 | G proteins | c.37.1.8 |
|  | 1foe_AB |  |  |  |  |
|  | 1ki1_AB |  |  |  |  |
|  | 1lb1_AB |  |  |  |  |
|  | 2dfk_AB |  |  |  |  |
|  | 1x86_AB |  |  |  |  |
| 17 | 2pv7_AB | TyrA dimerization domain-like | a.100.1.12 | TyrA dimerization domain-like | a.100.1.12 |
|  | 2g5c_AC |  |  |  |  |
|  | 2f1k_AB |  |  |  |  |
| 18 | 1tnb_AB | Protein prenyltransferases | a.102.4.3 | Protein prenylyltransferase | a.118.6.1 |
|  | 1ltx_AB |  |  |  |  |
|  | 1sa5_AB |  |  |  |  |
| 19 | 1aj8_AB | Citrate synthase | a.103.1.1 | Citrate synthase | a.103.1.1 |
|  | 1ixe_AB |  |  |  |  |
|  | 1o7x_AB |  |  |  |  |
|  | 4cts_AB |  |  |  |  |
|  | 1nxg_AB |  |  |  |  |
| 20 | 1dd4_AB | Ribosomal protein L7/12, oligomerisation (N-terminal) domain | a.108.1.1 | Ribosomal protein L7/12, oligomerisation (N-terminal) domain | a.108.1.1 |
|  | 1dd3_AB |  |  |  |  |
| 21 | 1s9d_AE | Sec7 domain | a.118.3.1 | G proteins | c.37.1.8 |
|  | 1re0_AB |  |  |  |  |
|  | 1r8q_AE |  |  |  |  |
| 22 | 1zk8_AB | Tetracyclin repressor-like, C-terminal domain | a.121.1.1 | Tetracyclin repressor-like, C-terminal domain | a.121.1.1 |
|  | 2gfn_AB |  |  |  |  |
|  | 2hku_AB |  |  |  |  |
|  | 1z0x_AB |  |  |  |  |
|  | 1vi0_AB |  |  |  |  |
|  | 2g3b_AB |  |  |  |  |
|  | 1rpw_AB |  |  |  |  |
|  | 2zoz_AB |  |  |  |  |
|  | 1rkt_AB |  |  |  |  |
|  | 1t33_AB |  |  |  |  |
|  | 3loc_AB |  |  |  |  |
|  | 1bjy_AB |  |  |  |  |
| 23 | 2gpp_AB | Nuclear receptor ligand-binding domain | a.123.1.1 | Nuclear receptor ligand-binding domain | a.123.1.1 |
|  | 1yy4_AB |  |  |  |  |
|  | 1h9u_AB |  |  |  |  |
|  | 1uhl_AB |  |  |  |  |
|  | 1m7w_AB |  |  |  |  |
|  | 1xls_AE |  |  |  |  |
|  | 1fm6_AD |  |  |  |  |
|  | 1r1k_AD |  |  |  |  |
|  | 1xdk_AB |  |  |  |  |
|  | 1zeo_AB |  |  |  |  |
|  | 1ot7_AB |  |  |  |  |
|  | 1p8d_AB |  |  |  |  |
| 24 | 1gk2_AD | HAL/PAL-like | a.127.1.2 | HAL/PAL-like | a.127.1.2 |
|  | 1w27_AB |  |  |  |  |
|  | 1t6p_AC |  |  |  |  |
| 25 | 2q80_AB | Isoprenyldiphosphate synthases | a.128.1.1 | Isoprenyldiphosphate synthases) | a.128.1.1 |
|  | 1v4e_AB |  |  |  |  |
|  | 1rtr_AB |  |  |  |  |
|  | 1rqi_AB |  |  |  |  |
| 26 | 2d8d_AB | Dimericchorismatemutase | a.130.1.1 | Dimericchorismatemutase | a.130.1.1 |
|  | 1ecm_AB |  |  |  |  |
|  | 2h9c_AB |  |  |  |  |
| 27 | 1wwm_AB | TENA/THI-4 | a.132.1.3 | TENA/THI-4 | a.132.1.3 |
|  | 1rtw_AB |  |  |  |  |
|  | 2gm8_AB |  |  |  |  |
|  | 1yaf_AC |  |  |  |  |
|  | 1udd_AB |  |  |  |  |
|  | 1z72_AB |  |  |  |  |
| 28 | 1cl5_AB | Vertebrate phospholipase A2 | a.133.1.2 | Vertebrate phospholipase A2 | a.133.1.2 |
|  | 1y38_AB |  |  |  |  |
|  | 1oyf_AB |  |  |  |  |
| 29 | 2ijc_AF | Atu0492-like | a.152.1.3 | Atu0492-like | a.152.1.3 |
|  | 2gmy_AB |  |  |  |  |
|  | 2oyo_AB |  |  |  |  |
|  | 2prr_AB |  |  |  |  |
|  | 2pfx_AB |  |  |  |  |
| 30 | 1fqv_AB | Skp1 dimerisation domain-like | a.157.1.1 | F-box domain | a.158.1.1 |
|  | 1nex_AB |  |  |  |  |
|  | 1p22_AB |  |  |  |  |
|  | 2ovr_AB |  |  |  |  |
| 31 | 1y74_AD | L27 domain | a.194.1.1 | L27 domain | a.194.1.1 |
|  | 1vf6_AC |  |  |  |  |
|  | 1y76_AD |  |  |  |  |
|  | 1rso_AB |  |  |  |  |
|  | 1zl8_AB |  |  |  |  |
| 32 | 2qgs_AB | HD domain | a.211.1.1 | (HD domain | a.211.1.1 |
|  | 2pjq_BC |  |  |  |  |
|  | 3dto_AB |  |  |  |  |
|  | 3djb_AB |  |  |  |  |
| 33 | 2jr2_AB | YejL-like | a.284.1.1 | YejL-like | a.284.1.1 |
|  | 2juz_AB |  |  |  |  |
|  | 2jrx_AB |  |  |  |  |
| 34 | 1n0l_AB | Pilus chaperone | b.1.11.1 | Pilus subunits | b.2.3.2 |
|  | 2uy7_AB |  |  |  |  |
|  | 1pdk_AB |  |  |  |  |
|  | 2j2z_AB |  |  |  |  |
|  | 1ze3_CH |  |  |  |  |
|  | 1p5u_AB |  |  |  |  |
|  | 2co6_AB |  |  |  |  |
| 35 | 1fft_AB | Periplasmic domain of cytochrome c oxidase subunit II | b.6.1.2 | Cytochrome c oxidase subunit I-like | f.24.1.1 |
|  | 1ehk_AB |  |  |  |  |
|  | 3dtu_AB |  |  |  |  |
|  | 2dys_AB |  |  |  |  |
| 36 | 1fi8_AC | Ecotin, trypsin inhibitor | b.16.1.1 | Eukaryotic proteases | b.47.1.2 |
|  | 1id5_HI |  |  |  |  |
|  | 1azz_AC |  |  |  |  |
|  | 1n8o_BE |  |  |  |  |
|  | 1xx9_AC |  |  |  |  |
|  | 1p0s_HE |  |  |  |  |
|  | 1ezu_BC |  |  |  |  |
| 37 | 1uxe_AB | Adenovirus fiber protein "knob" domain | b.21.1.1 | Adenovirus fiber protein "knob" domain | b.21.1.1 |
|  | 1qiu_AB |  |  |  |  |
|  | 1nob_AB |  |  |  |  |
| 38 | 1xu2_AR | TNF-like | b.22.1.1 | BAFF receptor-like | g.24.1.2 |
|  | 1oqd_AK |  |  |  |  |
|  | 1oqe_AK |  |  |  |  |
|  | 1xu1_AR |  |  |  |  |
| 39 | 1ukg_AB | Legume lectins | b.29.1.1 | Legume lectins | b.29.1.1 |
|  | 2dvg_AB |  |  |  |  |
|  | 1qmo_AE |  |  |  |  |
|  | 1qot_AB |  |  |  |  |
|  | 1avb_AB |  |  |  |  |
|  | 1fat_AB |  |  |  |  |
|  | 2ltn_AC |  |  |  |  |
| 40 | 1n9e_AB | Amine oxidase catalytic domain | b.30.2.1 | Amine oxidase catalytic domain | b.30.2.1 |
|  | 2oov_AB |  |  |  |  |
|  | 1ivu_AB |  |  |  |  |
|  | 1qal_AB |  |  |  |  |
|  | 1ksi_AB |  |  |  |  |
| 41 | 2b24_AB | Ring hydroxylating alpha subunit ISP domain | b.33.1.2 | Ring hydroxylating alpha subunit catalytic domain | d.129.3.3 |
|  | 1uli_AB |  |  |  |  |
|  | 1ndo_AB |  |  |  |  |
| 42 | 2cz0_AB | Nitrile hydratase beta chain | b.34.4.4 | Nitrile hydratase alpha chain | d.149.1.1 |
|  | 1v29_AB |  |  |  |  |
|  | 1ugr_AB |  |  |  |  |
| 43 | 1hx5_AB | GroES | b.35.1.1 | GroES | b.35.1.1 |
|  | 1wnr_AB |  |  |  |  |
|  | 1pf9_OP |  |  |  |  |
| 44 | 1n9s_AB | Sm motif of small nuclear ribonucleoproteins, SNRNP | b.38.1.1 | Sm motif of small nuclear ribonucleoproteins, SNRNP | b.38.1.1 |
|  | 1i8f_AB |  |  |  |  |
|  | 1d3b_AB |  |  |  |  |
|  | 1b34_AB |  |  |  |  |
|  | 1m8v_AB |  |  |  |  |
|  | 1i5l_AB |  |  |  |  |
|  | 1i4k_AB |  |  |  |  |
| 45 | 1lt5_DE | Bacterial AB5 toxins, B-subunits | b.40.2.1 | Bacterial AB5 toxins, B-subunits | b.40.2.1 |
|  | 1qb5_DE |  |  |  |  |
| 46 | 1ggp_AB | Ricin B-like | b.42.2.1 | Plant cytotoxins | d.165.1.1 |
|  | 2q3n_AB |  |  |  |  |
|  | 2mll_AB |  |  |  |  |
|  | 1hwn_AB |  |  |  |  |
|  | 2aai_AB |  |  |  |  |
| 47 | 1usc_AB | NADH:FMN oxidoreductase-like | b.45.1.2 | NADH:FMN oxidoreductase-like | b.45.1.2 |
|  | 1rz1_AB |  |  |  |  |
|  | 1i0r_AB |  |  |  |  |
| 48 | 1hkw_AB | Eukaryotic ODC-like | b.49.2.3 | Alanine racemase-like, N-terminal domain | c.1.6.1 |
|  | 1qu4_AB |  |  |  |  |
|  | 1twi_AB |  |  |  |  |
| 49 | 2rsp_AB | Retroviral protease (retropepsin) | b.50.1.1 | Retroviral protease (retropepsin) | b.50.1.1 |
|  | 1ivp_AB |  |  |  |  |
|  | 2p3b_AB |  |  |  |  |
|  | 1ec2_AB |  |  |  |  |
|  | 3fiv_AB |  |  |  |  |
| 50 | 1ytf_CD | Transcription factor IIA (TFIIA), beta-barrel domain | b.56.1.1 | Transcription factor IIA (TFIIA), beta-barrel domain | b.56.1.1 |
|  | 1nvp_CD |  |  |  |  |
|  | 1nh2_CD |  |  |  |  |
| 51 | 2pbk_AB | Herpes virus serine proteinase, assemblin | b.57.1.1 | Herpes virus serine proteinase, assemblin | b.57.1.1 |
|  | 1o6e_AB |  |  |  |  |
|  | 1at3_AB |  |  |  |  |
|  | 1id4_AB |  |  |  |  |
|  | 1nju_AB |  |  |  |  |
| 52 | 2pa7_AB | dTDP-sugar isomerase | b.82.1.1 | dTDP-sugar isomerase | b.82.1.1 |
|  | 1pm7_AB |  |  |  |  |
|  | 1ofn_AB |  |  |  |  |
|  | 1dzr_AB |  |  |  |  |
|  | 1wlt_AB |  |  |  |  |
|  | 2ixl_AB |  |  |  |  |
| 53 | 1f7p_AB | dUTPase-like | b.85.4.1 | dUTPase-like | b.85.4.1 |
|  | 1q5h_AB |  |  |  |  |
|  | 1smc_AB |  |  |  |  |
| 54 | 1gkq_AD | Hydantoinase (dihydropyrimidinase) | b.92.1.3 | Hydantoinase (dihydropyrimidinase) | b.92.1.3 |
|  | 1k1d_AB |  |  |  |  |
|  | 1gkr_AB |  |  |  |  |
|  | 2fvm_AC |  |  |  |  |
| 55 | 2vq0_AB | Tombusviridae-like VP | b.121.4.7 | Tombusviridae-like VP | b.121.4.7 |
|  | 1c8n_AB |  |  |  |  |
|  | 4sbv_AB |  |  |  |  |
|  | 1f2n_AB |  |  |  |  |
| 56 | 2nad_AB | (Formate/glycerate dehydrogenases, NAD-domain | c.2.1.4 | Formate/glycerate dehydrogenases, NAD-domain | c.2.1.4 |
|  | 1j49_AB |  |  |  |  |
|  | 1qp8_AB |  |  |  |  |
|  | 1ygy_AB |  |  |  |  |
|  | 1psd_AB |  |  |  |  |
|  | 1gdh_AB |  |  |  |  |
| 57 | 1z7x_WX | 28-residue LRR | c.10.1.1 | Ribonuclease A-like | d.5.1.1 |
|  | 1a4y_AB |  |  |  |  |
|  | 2bex_AC |  |  |  |  |
| 58 | 1yg8_AG | Clp protease, ClpP subunit | c.14.1.1 | Clp protease, ClpP subunit | c.14.1.1 |
|  | 2ce3_AG |  |  |  |  |
|  | 1y7o_AG |  |  |  |  |
|  | 1tg6_AG |  |  |  |  |
|  | 2f6i_AG |  |  |  |  |
| 59 | 1lqm_AB | Uracil-DNA glycosylase | c.18.1.1 | (Uracil-DNA glycosylase inhibitor protein | d.17.5.1 |
|  | 2j8x_AB |  |  |  |  |
|  | 1udi_EI |  |  |  |  |
| 60 | 2c57_AB | Type II 3-dehydroquinate dehydratase | c.23.13.1 | Type II 3-dehydroquinate dehydratase | c.23.13.1 |
|  | 1gqo_AB |  |  |  |  |
|  | 1v1j_AB |  |  |  |  |
| 61 | 1ufv_AB | Pantothenatesynthetase (Pantoate-beta-alanine ligase, PanC)) | c.26.1.4 | Pantothenatesynthetase (Pantoate-beta-alanine ligase, PanC) | c.26.1.4 |
|  | 1iho_AB |  |  |  |  |
|  | 3coy_AB |  |  |  |  |
| 62 | 1zpd_AB | Pyruvate oxidase and decarboxylase Pyr module | c.36.1.5 | Pyruvate oxidase and decarboxylase PP module | c.36.1.9 |
|  | 1pyd_AB |  |  |  |  |
|  | 1ozh_AB |  |  |  |  |
|  | 1ovm_AB |  |  |  |  |
|  | 2c31_AB |  |  |  |  |
|  | 1upb_AB |  |  |  |  |
|  | 1n0h_AB |  |  |  |  |
|  | 1mcz_AB |  |  |  |  |
| 63 | 1hoo_AB | Nitrogenase iron protein-like | c.37.1.10 | Nitrogenase iron protein-like | c.37.1.10 |
|  | 1lny_AB |  |  |  |  |
|  | 1dj3_AB |  |  |  |  |
| 64 | 2tec_EI | Subtilases | c.41.1.1 | CI-2 family of serine protease inhibitors | d.40.1.1 |
|  | 1mee_AI |  |  |  |  |
|  | 1y3f_EI |  |  |  |  |
| 65 | 1nw2_AD | Thioltransferase | c.47.1.1 | Thioltransferase | c.47.1.1 |
|  | 1xwb_CD |  |  |  |  |
|  | 1ep8_AB |  |  |  |  |
|  | 1f9m_AB |  |  |  |  |
| 66 | 1a49_AB | Pyruvate kinase, C-terminal domain | c.49.1.1 | Pyruvate kinase, C-terminal domain | c.49.1.1 |
|  | 1pky_AB |  |  |  |  |
|  | 1pkl_AB |  |  |  |  |
| 67 | 1g5c_AB | beta-carbonic anhydrase, cab | c.53.2.1 | beta-carbonic anhydrase, cab | c.53.2.1 |
|  | 2esf_AB |  |  |  |  |
|  | 1ekj_AB |  |  |  |  |
| 68 | 2nrh_AB | CoaX-like | c.55.1.13 | CoaX-like | c.55.1.13 |
|  | 2f9w_AB |  |  |  |  |
|  | 3bf1_AB |  |  |  |  |
| 69 | 1je1_AD | Purine and uridinephosphorylases | c.56.2.1 | Purine and uridinephosphorylases | c.56.2.1 |
|  | 1k9s_AD |  |  |  |  |
|  | 1odi_AB |  |  |  |  |
|  | 1nw4_AB |  |  |  |  |
|  | 1tgv_AB |  |  |  |  |
|  | 1ybf_AB |  |  |  |  |
|  | 1t8w_AB |  |  |  |  |
| 70 | 1bxg_AB | Aminoacid dehydrogenases | c.58.1.1 | Aminoacid dehydrogenases | c.58.1.1 |
|  | 1leh_AB |  |  |  |  |
|  | 1bvu_AF |  |  |  |  |
|  | 1v9l_AF |  |  |  |  |
|  | 1nr7_AE |  |  |  |  |
| 71 | 1bq3_AB | Cofactor-dependent phosphoglycerate mutase | c.60.1.1 | Cofactor-dependent phosphoglycerate mutase | c.60.1.1 |
|  | 2a9j_AB |  |  |  |  |
|  | 1rii_AB |  |  |  |  |
| 72 | 1a96_AB | Phosphoribosyltransferases (PRTases) | c.61.1.1 | Phosphoribosyltransferases (PRTases) | c.61.1.1 |
|  | 1vdm_AB |  |  |  |  |
|  | 1pzm_AB |  |  |  |  |
|  | 1hgx_AB |  |  |  |  |
|  | 1grv_AB |  |  |  |  |
|  | 1tc1_AB |  |  |  |  |
|  | 1hmp_AB |  |  |  |  |
|  | 1cjb_AB |  |  |  |  |
|  | 1qk4_AB |  |  |  |  |
| 73 | 1iy9_AD | Spermidine synthase | c.66.1.17 | Spermidine synthase | c.66.1.17 |
|  | 2o0l_AB |  |  |  |  |
|  | 1mjf_AB |  |  |  |  |
|  | 1uir_AB |  |  |  |  |
|  | 2b2c_AB |  |  |  |  |
|  | 2q41_AD |  |  |  |  |
|  | 1jq3_AD |  |  |  |  |
| 74 | 1cs1_AD | Cystathionine synthase-like | c.67.1.3 | Cystathionine synthase-like | c.67.1.3 |
|  | 1gc0_AB |  |  |  |  |
|  | 1n8p_AD |  |  |  |  |
|  | 1i43_AD |  |  |  |  |
| 75 | 1h3m_AB | Cytidylytransferase | c.68.1.13 | Cytidylytransferase | c.68.1.13 |
|  | 1vpa_AB |  |  |  |  |
|  | 1vgw_AB |  |  |  |  |
| 76 | 2abq_AB | Ribokinase-like | c.72.1.1 | Ribokinase-like | c.72.1.1 |
|  | 2awd_AB |  |  |  |  |
|  | 2ajr_AB |  |  |  |  |
|  | 2dcn_AB |  |  |  |  |
|  | 1tz3_AB |  |  |  |  |
|  | 1gqt_AB |  |  |  |  |
|  | 1v19_AB |  |  |  |  |
|  | 2afb_AB |  |  |  |  |
|  | 1vm7_AB |  |  |  |  |
| 77 | 2bri_AB | PyrH-like | c.73.1.3 | PyrH-like | c.73.1.3 |
|  | 2brx_AB |  |  |  |  |
|  | 2v4y_AF |  |  |  |  |
| 78 | 1gc8_AB | Dimericisocitrate&isopropylmalate dehydrogenases | c.77.1.1 | Dimericisocitrate&isopropylmalate dehydrogenases | c.77.1.1 |
|  | 1wpw_AB |  |  |  |  |
|  | 1a05_AB |  |  |  |  |
|  | 1t0l_AB |  |  |  |  |
|  | 2g4o_AB |  |  |  |  |
|  | 1hqs_AB |  |  |  |  |
| 79 | 2bht_AB | (Tryptophan synthase beta subunit-like PLP-dependent enzymes | c.79.1.1 | Tryptophan synthase beta subunit-like PLP-dependent enzymes | c.79.1.1 |
|  | 1o58_AB |  |  |  |  |
|  | 1oas_AB |  |  |  |  |
|  | 1pwh_AB |  |  |  |  |
|  | 1tzm_AB |  |  |  |  |
|  | 1ve5_AD |  |  |  |  |
|  | 1j0b_AB |  |  |  |  |
|  | 1v7c_AB |  |  |  |  |
|  | 1m54_AB |  |  |  |  |
|  | 1wdw_BD |  |  |  |  |
|  | 1wkv_AB |  |  |  |  |
|  | 1e5x_AB |  |  |  |  |
| 80 | 1viv_AB | mono-SIS domain | c.80.1.3 | mono-SIS domain | c.80.1.3 |
|  | 1vim_AB |  |  |  |  |
|  | 3bjz_AB |  |  |  |  |
|  | 1x94_AB |  |  |  |  |
|  | 1tk9_AD |  |  |  |  |
| 81 | 1vle_MN | Formate dehydrogenase/DMSO reductase, domains 1-3 | c.81.1.1 | Ferredoxin domains from multidomain proteins | d.58.1.5 |
|  | 1kqg_AB |  |  |  |  |
|  | 1h0h_AB |  |  |  |  |
| 82 | 1ad3_AB | ALDH-like | c.82.1.1 | ALDH-like | c.82.1.1 |
|  | 1wnd_AD |  |  |  |  |
|  | 1euh_AB |  |  |  |  |
|  | 1ez0_AD |  |  |  |  |
|  | 1o02_AB |  |  |  |  |
|  | 1uzb_AB |  |  |  |  |
|  | 1bpw_AB |  |  |  |  |
| 83 | 1vgv_AB | UDP-N-acetylglucosamine 2-epimerase | c.87.1.3 | UDP-N-acetylglucosamine 2-epimerase | c.87.1.3 |
|  | 1v4v_AB |  |  |  |  |
|  | 1o6c_AB |  |  |  |  |
| 84 | 1jja_AC | Glutaminase/Asparaginase | c.88.1.1 | Glutaminase/Asparaginase | c.88.1.1 |
|  | 1hfw_AC |  |  |  |  |
|  | 2ocd_AB |  |  |  |  |
|  | 1djo_AB |  |  |  |  |
|  | 2d6f_AB |  |  |  |  |
| 85 | 1jhz_AB | L-arabinose binding protein-like | c.93.1.1 | L-arabinose binding protein-like | c.93.1.1 |
|  | 1lbi_AB |  |  |  |  |
|  | 1sxi_AD |  |  |  |  |
|  | 1byk_AB |  |  |  |  |
| 86 | 1mm6_AB | Phosphate binding protein-like) | c.94.1.1 | Phosphate binding protein-like | c.94.1.1 |
|  | 2a5t_AB |  |  |  |  |
|  | 1y1m_AB |  |  |  |  |
| 87 | 1u0m_AB | Chalcone synthase-like | c.95.1.2 | Chalcone synthase-like | c.95.1.2 |
|  | 1ub7_AB |  |  |  |  |
|  | 1xpk_AB |  |  |  |  |
|  | 2eft_AB |  |  |  |  |
|  | 1tee_AB |  |  |  |  |
|  | 1mzj_AB |  |  |  |  |
|  | 1u0u_AB |  |  |  |  |
|  | 2qnz_AB |  |  |  |  |
| 88 | 1ux1_AD | Cytidine deaminase | c.97.1.1 | Cytidine deaminase | c.97.1.1 |
|  | 2z3h_AD |  |  |  |  |
|  | 2fr6_AD |  |  |  |  |
|  | 1r5t_AC |  |  |  |  |
| 89 | 1vhy_AB | YggJ C-terminal domain-like | c.116.1.5 | YggJ C-terminal domain-like | c.116.1.5 |
|  | 1v6z_AB |  |  |  |  |
|  | 1vhk_AB |  |  |  |  |
| 90 | 2h1o_AB | PIN domain | c.120.1.1 | PIN domain | c.120.1.1 |
|  | 1v8o_AB |  |  |  |  |
|  | 1v96_AB |  |  |  |  |
| 91 | 1vgq_AB | CoA-transferase family III (CaiB/BaiF) | c.123.1.1 | CoA-transferase family III (CaiB/BaiF) | c.123.1.1 |
|  | 1x74_AB |  |  |  |  |
|  | 1xa3_AB |  |  |  |  |
| 92 | 1poi_AC | CoA transferase alpha subunit-like | c.124.1.2 | CoA transferase alpha subunit-like | c.124.1.2 |
|  | 1ope_AB |  |  |  |  |
|  | 1k6d_AB |  |  |  |  |
|  | 2ahu_AD |  |  |  |  |
| 93 | 2g4d_AB | Adenain-like | d.3.1.7 | Ubiquitin-related | d.15.1.1 |
|  | 2bkr_AB |  |  |  |  |
|  | 2ckh_AB |  |  |  |  |
|  | 1euv_AB |  |  |  |  |
| 94 | 2z7a_AB | Ketosteroidisomerase-like | d.17.4.3 | Ketosteroidisomerase-like | d.17.4.3 |
|  | 1ohs_AB |  |  |  |  |
|  | 1cqs_AB |  |  |  |  |
| 95 | 1qy9_AB | PhzC/PhzF-like | d.21.1.2 | PhzC/PhzF-like | d.21.1.2 |
|  | 1u1w_AB |  |  |  |  |
|  | 1u0k_AB |  |  |  |  |
| 96 | 2i7r_AB | Antibiotic resistance proteins | d.32.1.2 | Antibiotic resistance proteins | d.32.1.2 |
|  | 2a4x_AB |  |  |  |  |
|  | 1ecs_AB |  |  |  |  |
|  | 2pjs_AB |  |  |  |  |
|  | 1xrk_AB |  |  |  |  |
|  | 1r9c_AB |  |  |  |  |
|  | 1nki_AB |  |  |  |  |
| 97 | 2h4u_AB | PaaI/YdiI-like | d.38.1.5 | PaaI/YdiI-like | d.38.1.5 |
|  | 1wm6_AC |  |  |  |  |
|  | 2f3x_AB |  |  |  |  |
|  | 1t82_AB |  |  |  |  |
|  | 1yoc_AB |  |  |  |  |
|  | 2fs2_AB |  |  |  |  |
|  | 1sh8_AB |  |  |  |  |
|  | 1sbk_AC |  |  |  |  |
|  | 1q4s_AB |  |  |  |  |
|  | 2ov9_AB |  |  |  |  |
| 98 | 1ues_AB | Fe,Mn superoxide dismutase (SOD), C-terminal domain | d.44.1.1 | Fe,Mn superoxide dismutase (SOD), C-terminal domain | d.44.1.1 |
|  | 2nyb_AB |  |  |  |  |
|  | 1bsm_AB |  |  |  |  |
|  | 1kkc_AB |  |  |  |  |
|  | 1wb8_AB |  |  |  |  |
| 99 | 1tkk_AF | Enolase N-terminal domain-like | d.54.1.1 | Enolase N-terminal domain-like | d.54.1.1 |
|  | 2dw6_AB |  |  |  |  |
|  | 3muc_AB |  |  |  |  |
|  | 2gl5_AB |  |  |  |  |
|  | 1sjd_AB |  |  |  |  |
|  | 1wue_AB |  |  |  |  |
|  | 1wuf_AB |  |  |  |  |
|  | 1yey_AB |  |  |  |  |
|  | 1r0m_AC |  |  |  |  |
| 100 | 1r3n_AB | Bacterial exopeptidase dimerisation domain | d.58.19.1 | Bacterial exopeptidase dimerisation domain | d.58.19.1 |
|  | 1vgy_AB |  |  |  |  |
|  | 1ysj_AB |  |  |  |  |
|  | 1vix_AB |  |  |  |  |
|  | 1cg2_AD |  |  |  |  |
|  | 1z2l_AB |  |  |  |  |
| 101 | 2p92_AB | TM1457-like | d.64.2.1 | TM1457-like | d.64.2.1 |
|  | 1s12_AD |  |  |  |  |
|  | 2g0j_AB |  |  |  |  |
|  | 2idl_AB |  |  |  |  |
| 102 | 1nfh_AB | DNA-binding protein AlbA | d.68.6.1 | DNA-binding protein AlbA | d.68.6.1 |
|  | 2bky_AX |  |  |  |  |
|  | 1udv_AB |  |  |  |  |
| 103 | 1xho_AB | Chorismate mutase | d.79.1.2 | Chorismate mutase | d.79.1.2 |
|  | 1com_AB |  |  |  |  |
|  | 1ode_AB |  |  |  |  |
| 104 | 1u1i_AB | Dihydrodipicolinatereductase-like | d.81.1.3 | Dihydrodipicolinatereductase-like | d.81.1.3 |
|  | 1arz_AD |  |  |  |  |
|  | 1jkf_AB |  |  |  |  |
|  | 1p1k_AB |  |  |  |  |
|  | 1yl7_AB |  |  |  |  |
|  | 1vm6_AD |  |  |  |  |
|  | 1r0l_AB |  |  |  |  |
|  | 3dap_AB |  |  |  |  |
| 105 | 1rm6_AB | CO dehydrogenase flavoprotein C-terminal domain-like | d.87.2.1 | Molybdenum cofactor-binding domain | d.133.1.1 |
|  | 1t3q_BC |  |  |  |  |
|  | 1jrp_AB |  |  |  |  |
|  | 1ffv_BC |  |  |  |  |
| 106 | 2b67_AB | NADH oxidase/flavinreductase | d.90.1.1 | NADH oxidase/flavinreductase | d.90.1.1 |
|  | 1oon_AB |  |  |  |  |
|  | 2ifa_AB |  |  |  |  |
|  | 1f5v_AB |  |  |  |  |
|  | 1v5y_AB |  |  |  |  |
|  | 2fre_AB |  |  |  |  |
| 107 | 1j2g_AD | Urate oxidase (uricase) | d.96.1.4 | Urate oxidase (uricase) | d.96.1.4 |
|  | 1xxj_AC |  |  |  |  |
|  | 2yzd_AB |  |  |  |  |
|  | 1vax_AB |  |  |  |  |
|  | 1ws3_AC |  |  |  |  |
| 108 | 1y4o_AB | Roadblock/LC7 domain | d.110.7.1 | Roadblock/LC7 domain | d.110.7.1 |
|  | 1veu_AB |  |  |  |  |
|  | 1j3w_AB |  |  |  |  |
| 109 | 1vhg_AB | MutT-like | d.113.1.1 | MutT-like | d.113.1.1 |
|  | 1viu_AB |  |  |  |  |
|  | 1viq_BC |  |  |  |  |
| 110 | 1syn_AB | Thymidylate synthase/dCMPhydroxymethylase | d.117.1.1 | Thymidylate synthase/dCMPhydroxymethylase | d.117.1.1 |
|  | 1b49_AC |  |  |  |  |
|  | 1f28_AB |  |  |  |  |
|  | 1bsp_AB |  |  |  |  |
|  | 1qzf_AB |  |  |  |  |
| 111 | 1kij_AB | DNA gyrase/MutL, N-terminal domain | d.122.1.2 | DNA gyrase/MutL, N-terminal domain | d.122.1.2 |
|  | 1mx0_AB |  |  |  |  |
|  | 1qzr_AB |  |  |  |  |
|  | 1s16_AB |  |  |  |  |
|  | 1ei1_AB |  |  |  |  |
| 112 | 1m35_AD | Creatinase/aminopeptidase | d.127.1.1 | Creatinase/aminopeptidase | d.127.1.1 |
|  | 1pv9_AB |  |  |  |  |
|  | 1chm_AB |  |  |  |  |
| 113 | 2qlv_AB | Ssp2 C-terminal domain-like | d.129.6.2 | AMPKBI-like | d.353.1.1 |
|  | 2v92_AB |  |  |  |  |
|  | 2ooy_AB |  |  |  |  |
| 114 | 2gac_AB | (Glycosyl)asparaginase | d.153.1.5 | (Glycosyl)asparaginase | d.153.1.5 |
|  | 1k2x_AB |  |  |  |  |
|  | 1apz_AB |  |  |  |  |
| 115 | 1sb2_AB | C-type lectin domain | d.169.1.1 | C-type lectin domain | d.169.1.1 |
|  | 1ukm_AB |  |  |  |  |
|  | 1x2w_AB |  |  |  |  |
|  | 1fvu_AB |  |  |  |  |
|  | 1uex_AB |  |  |  |  |
|  | 1oz7_AB |  |  |  |  |
|  | 1v4l_AB |  |  |  |  |
| 116 | 1inn_AB | Autoinducer-2 production protein LuxS | d.185.1.2 | Autoinducer-2 production protein LuxS | d.185.1.2 |
|  | 1j6w_AB |  |  |  |  |
|  | 1j6x_AB |  |  |  |  |
| 117 | 2pkh_AB | UTRA domain | d.190.1.2 | UTRA domain | d.190.1.2 |
|  | 3bwg_AB |  |  |  |  |
|  | 2ikk_AB |  |  |  |  |
|  | 2ooi_AB |  |  |  |  |
|  | 3ddv_AB |  |  |  |  |
|  | 2fa1_AB |  |  |  |  |
|  | 2p19_AC |  |  |  |  |
|  | 3cnv_AB |  |  |  |  |
| 118 | 2onf_AB | Ohr/OsmC resistance proteins | d.227.1.1 | Ohr/OsmC resistance proteins | d.227.1.1 |
|  | 1ukk_AB |  |  |  |  |
|  | 1qwi_AB |  |  |  |  |
|  | 1lql_AB |  |  |  |  |
|  | 1n2f_AB |  |  |  |  |
|  | 2d7v_AB |  |  |  |  |
|  | 2opl_AB |  |  |  |  |
| 119 | 1twj_AB | PurS subunit of FGAM synthetase | d.284.1.1 | PurS subunit of FGAM synthetase | d.284.1.1 |
|  | 1gtd_AB |  |  |  |  |
|  | 1vq3_AB |  |  |  |  |
| 120 | 1si8_AC | Heme-dependent catalases | e.5.1.1 | Heme-dependent catalases | e.5.1.1 |
|  | 1m7s_AC |  |  |  |  |
|  | 1a4e_AB |  |  |  |  |
|  | 1th2_AC |  |  |  |  |
|  | 1ggj_AC |  |  |  |  |
| 121 | 1dk4_AB | Inositol monophosphatase/fructose-1,6-bisphosphatase-like | e.7.1.1 | Inositol monophosphatase/fructose-1,6-bisphosphatase-like | e.7.1.1 |
|  | 1vdw_AB |  |  |  |  |
|  | 1lbv_AB |  |  |  |  |
|  | 1imd_AB |  |  |  |  |
| 122 | 2e75_AD | ISP transmembrane anchor | f.23.12.1 | Cytochrome b of cytochrome bc1 complex (Ubiquinol-cytochrome c reductase) | f.21.1.2 |
|  | 1q90_BR |  |  |  |  |
|  | 2ibz_CE |  |  |  |  |
|  | 2bcc_CE |  |  |  |  |
| 123 | 2axt_AD | Bacterial photosystem II reaction centre, L and M subunits | f.26.1.1 | Bacterial photosystem II reaction centre, L and M subunits | f.26.1.1 |
|  | 2gmr_LM |  |  |  |  |
|  | 1qov_LM |  |  |  |  |
|  | 6prc_LM |  |  |  |  |
| 124 | 1pdg_AB | Platelet-derived growth factor-like | g.17.1.1 | Platelet-derived growth factor-like | g.17.1.1 |
|  | 1bj1_VW |  |  |  |  |
|  | 1rv6_VW |  |  |  |  |
|  | 1wq9_AB |  |  |  |  |
|  | 1vpp_VW |  |  |  |  |
|  | 1kat_VW |  |  |  |  |

**Additional analysis on the SCOPPI data set**

In addition to the low-resolution data set described in the main text, we also performed the same analysis to an alternative low-resolution data set generated as follows. We took the original 609 protein dimers, and performed all-atomic molecular dynamics on them for 100ps at 300K. We then extracted the coordinates of the Cα atoms from the structural models corresponding to the last snapshot of each trajectory. The resulting models deviate from the original structural models by an average RMSD of 1.95 Å based on the Cα atoms. For this low-quality data set, our classification analysis resulted in an AUC of 0.924 for the ROC curve, which is still reasonable, again demonstrating the robustness of our method.

Furthermore, we would like to see if there is any bias of the three methods toward identifying similarity in homodimer interfaces or heterodimer interfaces. We partitioned the original SCOPPI data set into one containing homodimers only (480) and the other containing heterodimers only (129). With the same analysis described in the main text, the ROC curve generated by PCalign gives an AUC of 0.959 for the homodimers set, and 0.998 for the heterodimers set. For Ialign, this is 0.973 for the homodimers set and 0.997 for the heterodimers set respectively. The ROC curves produced by I2isiteengine yield an AUC of 0.818 for the homodimers set and 0.869 for the heterodimers set with their match score, an AUC of 0.884 for the homodimers set and 0.884 for the heterodimers set with their total score, and an AUC of 0.904 for the homodimers set and 0.924 for the heterodimers set with their t score. Thus all three methods perform better on the heterodimers set.

**Computational costs of different interface comparison methods**:

For comparison of computational time among the different methods, we collected the statistics of the running time reported by each program for the 185136 pairs we compared, excluding the one-time cost for preparing the dataset in the cases of PCalign and I2I-SiteEngine. Our performance lies between that of Ialign and I2I-SiteEngine, as shown in the boxplot of **Fig. S6**.


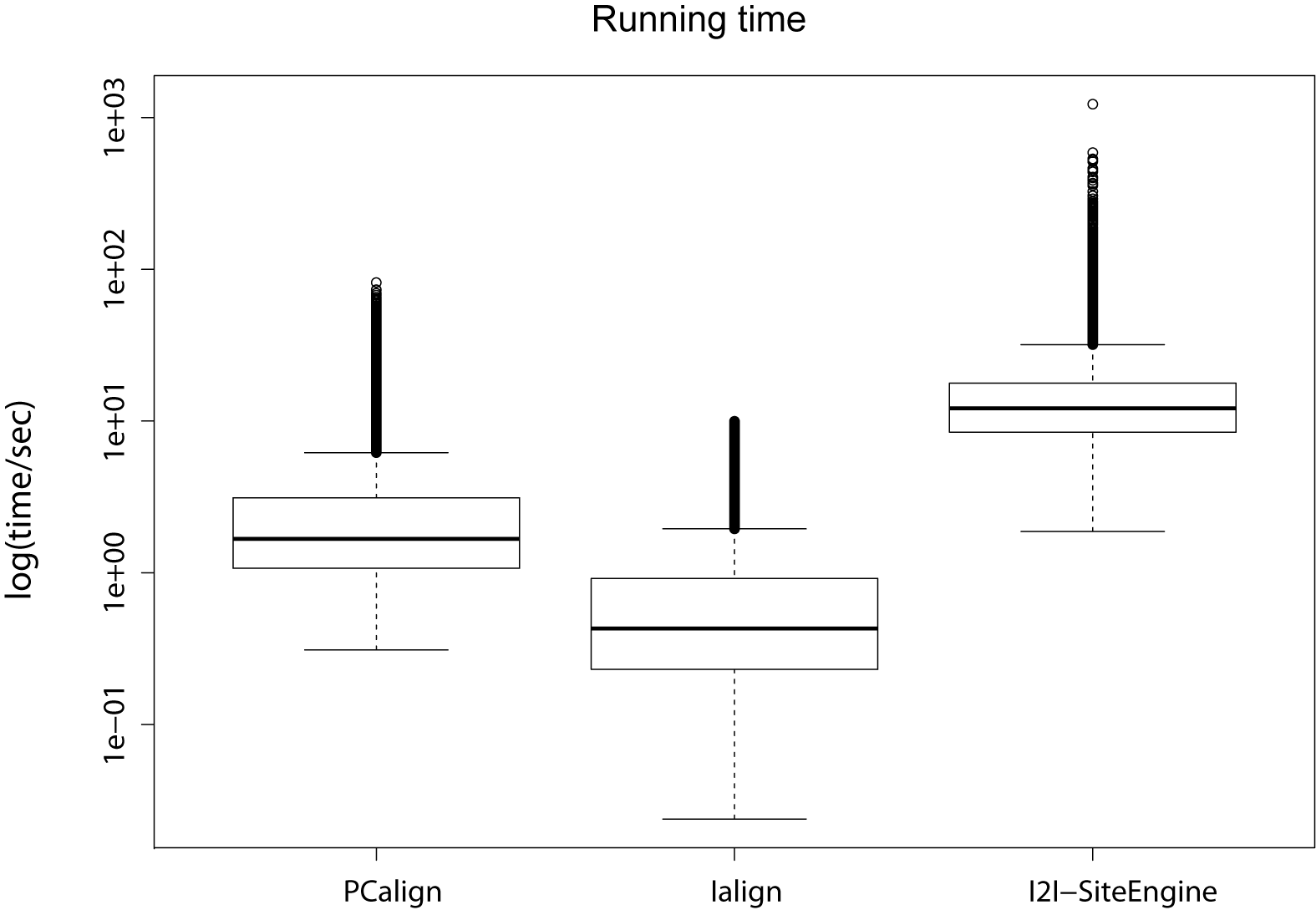


**Fig. S6**. Running time reported by the three programs. The time in seconds is plotted in log scale. PCalign finishes a comparison within a second on average, whereas Ialign completes within a fraction of a second. I2I-SiteEngine is roughly an order of magnitude slower. The longest computational time for these methods is spent on comparing (single-domained) dimers with over 200 interfacial residues, shown in circles on the very top of each box.

Supplemental Table 6. Three examples of viral mimicry achieved via convergent evolution. The first seven columns describe the identity of the proteins involved as well as their representative PDB IDs and corresponding chains used for our analysis. The eighth column gives the sequence identity between each viral protein and the endogenous binding partner it displaces, using the program ClustalW ([Li, 2003](#_ENREF_4)). The ninth column gives the template modeling score (TM-score) between each viral protein and the endogenous binding partner it mimics, where TM-score measures the structural similarity between two proteins ([Zhang and Skolnick, 2004](#_ENREF_5)), and a TM-score of 0.4 or higher typically implies high structural similarity. The last column corresponds to the PC-score of the two interfaces measured by our program, and a PC-score of 0.4 or higher implies highly similar interfaces. In all three examples, the cognate binding partner of the target protein shares no sequence or structural similarity with the viral protein, as evident from the low sequence identity and TM-scores. In all three cases, PCalign computes a significantly high score for the two interfaces, recognizing the interfacial patterns that the viral proteins evolved to mimic over time. We also compute the interface similarity scores returned by the two existing measures. For Ialign, the non-sequential version and not the sequential version recognizes the convergently evolved interfaces as significantly similar with IS-scores of 0.393, 0.436 and 0.299 for the three cases respectively. For I2I-SiteEngine, we had no success generating the input files for the first case of M3 protein for which the program reported an error. For the remaining two cases, the match scores are 27 and 22, the total scores 1339 and 648, and the t-scores 47 and 44 respectively. While it is not clear whether these scores are statistically significant, a comparison with the large scale study performed on the SCOPPI benchmark dataset suggests that these scores are not sufficient (the latter suggest a match score of at least 31 to be classified as similar interfaces).

| Viral protein | PDB ID | Human target protein | PDB ID (complexed with viral protein) | PDB ID (complexed with cognate partner) | Displaced human binding partner | PDB ID | Seq identity | TM-score | PC-score |
| --- | --- | --- | --- | --- | --- | --- | --- | --- | --- |
| Murid herpesvirus 4, M3 | 2nz1A | C-C motif chemokine 2 | 2nz1D | 1dokA | C-C motif chemokine 2 | 1dokB | 7% | 0.17940 | 0.445 |
| Simian virus 5, nonstructural protein V | 2b5lC | DNA damage-binding protein 1 | 2b5lA | 3ei4A | DNA damage-binding protein 2 | 3ei4B | 4% | 0.21976 | 0.546 |
| Nipah virus, glycoprotein G | 2vskA | Ephrin-B2 | 2vskB | 2hleB | Ephrin type-B receptor 4 | 2hleA | 12% | 0.24913 | 0.430 |

**Supplemental references**:

Damodaran, K.V.*, et al.* (2002) A general method to quantify quasi-equivalence in icosahedral viruses, *Journal of molecular biology*, **324**, 723-737.

Gao, M. and Skolnick, J. (2010) iAlign: a method for the structural comparison of protein-protein interfaces, *Bioinformatics*, **26**, 2259-2265.

Kuhn, H.W. (2005) The Hungarian Method for the assignment problem, *Nav Res Log*, **52**, 7-21.

Li, K.B. (2003) ClustalW-MPI: ClustalW analysis using distributed and parallel computing, *Bioinformatics*, **19**, 1585-1586.

Zhang, Y. and Skolnick, J. (2004) Scoring function for automated assessment of protein structure template quality, *Proteins*, **57**, 702-710.
